# Highly efficient manipulation of nervous system gene expression with NEPTUNE

**DOI:** 10.1016/j.crmeth.2021.100043

**Published:** 2021-07-06

**Authors:** Katrin Mangold, Jan Mašek, Jingyan He, Urban Lendahl, Elaine Fuchs, Emma R. Andersson

**Affiliations:** 1 Department of Cell and Molecular Biology, Karolinska Institutet, Stockholm 17177, Sweden; 2 Department of Biosciences and Nutrition, Karolinska Institutet, Huddinge 14183, Sweden; 3 Laboratory of Mammalian Cell Biology and Development, Howard Hughes Medical Institute, The Rockefeller University, New York, NY 10065, USA

**Keywords:** brain, spinal cord, genetic manipulation, mouse models, neurulation, lentivirus, Olig2, Sptbn2, *in utero* nano-injection, conditional

## Abstract

Genetic loss and gain of function in mice have typically been studied by using knockout or knockin mice that take months to years to generate. To address this problem for the nervous system, we developed NEPTUNE (NEural Plate Targeting by *in Utero* NanoinjEction) to rapidly and flexibly transduce the neural plate with virus prior to neurulation, and thus manipulate the future nervous system. Stable integration in >95% of cells in the brain enabled long-term overexpression, and conditional expression was achieved by using cell-type-specific MiniPromoters. Knockdown of *Olig2* by using NEPTUNE recapitulated the phenotype of *Olig2*^−/−^ embryos. We used NEPTUNE to investigate *Sptbn2*, mutations in which cause spinocerebellar ataxia type 5. *Sptbn2* knockdown induced dose-dependent defects in the neural tube, embryonic turning, and abdominal wall closure, previously unreported functions for *Sptbn2*. NEPTUNE thus offers a rapid and cost-effective technique to test gene function in the nervous system and can reveal phenotypes incompatible with life.

## Highlights

• *In utero* mouse neural plate viral transduction targets >95% of the developing brain
• Conditional NEPTUNE of neurons, astrocytes, and oligodendrocytes with MiniPromoters
• *Olig2* NEPTUNE knockdown recapitulates *Olig2*^−/−^ mice
• *Sptbn2* NEPTUNE knockdown reveals embryonic turning and abdominal wall defects

## Motivation

Current methods used to alter gene expression in mouse embryos to study brain development lack efficacy and are time consuming. NEPTUNE provides an addition to the molecular toolbox, allowing rapid and widespread manipulation of gene expression before neurulation *in vivo*. This technique can be adapted to specifically target central nervous system (CNS) cell subtypes such as neurons or glia. Functional analysis can be performed within days after injection. NEPTUNE can thus reduce the financial and ethical costs of animal research, while accelerating the process from hypothesis to results *in vivo*.

## Graphical abstract

**Figure:**
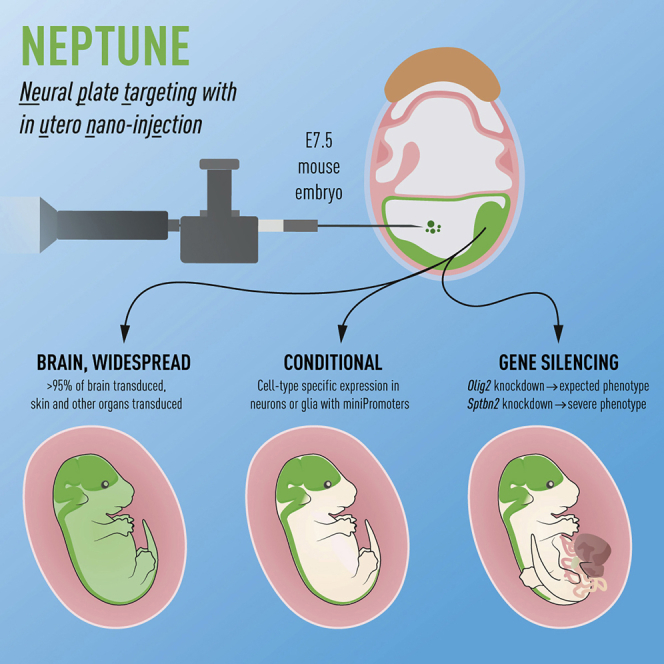

## Teaser

Mangold et al. present NEPTUNE: delivery of high-titer lentivirus to mouse embryos prior to brain formation, allowing manipulation of gene expression from initiation of nervous system development. Brain targeting is >95% cell type specific with MiniPromoters, stable for at least 6 months, recapitulates published phenotypes, and reveals embryonic functions for *Sptbn2*.

## Introduction

Recent progress in genomics, metagenomics, and transcriptomics has accelerated the identification of gene variants associated with human disease (Chiu and Miller, 2019; Taylor et al., 2015). *In vivo* validation in mammalian systems has, however, lagged behind, given that generation of mouse models is limited by the long reproduction time of mice. Ultrasound-guided *in utero* transduction with fluorescently traceable lentiviruses, carrying RNAi or Cre recombinase, into mouse embryos demonstrated noninvasive and highly efficient transduction of surface epithelium (Beronja et al., 2010, 2013) allowing rapid deciphering of gene networks in skin (Adam et al., 2015; Schramek et al., 2014). In contrast, genetic manipulation of embryonic mouse brain has largely relied on *in utero* electroporation (Saito and Nakatsuji, 2001; Tabata and Nakajima, 2001) or viral infection (Artegiani and Calegari, 2013) post neurulation, which targets fewer cells and cannot achieve long-term genetic manipulation, nor systemic effects.

The neural plate, prior to the onset of neurulation, is a single layer of columnar cells exposed to the amniotic fluid. We hypothesized that *in utero* injection at this stage, embryonic day 7.5 (E7.5) in mice, should achieve widespread transduction of the future brain and spinal cord. Injection of fluorescent beads into amniotic fluid at E7.5 is well-tolerated by mouse embryos (Slevin et al., 2006) and injection at E8.5 can target the future brain (Gaiano et al., 1999), suggesting that viral transduction at E7.5 could offer a significant improvement in targeting efficiency. Injections of minute virus of mice (MVM) at E9.5–E12.5 (Itah et al., 2004), or recombinant adeno-associated virus 2 (rAAV) at E15 (Lipshutz et al., 2001), were well-tolerated. However, only 41% of embryos injected with retrovirus at E8.5 survived, and of these 41% displayed exencephaly (Gaiano et al., 1999), indicating that injections at E8.5 with virus either is detrimental to development or requires further technical development.

Embryonic virus injection can achieve widespread transduction of most organs (Lipshutz et al., 2001); therefore, a method to achieve cell-type-specific expression would be desirable for targeting the nervous system exclusively. Conditional CRE expression to excise *LoxP*-flanked genetic sequences is a gold standard for spatiotemporal cell-type-specific expression but is associated with adverse effects related to random *Cre* integration, unpredictable genome editing, and toxic effects of CRE itself (Cain-Hom et al., 2017; Loonstra et al., 2001; Pomplun et al., 2007). An approach avoiding the use of dedicated Cre mouse strains is the use of MiniPromoters (Portales-Casamar et al., 2010) to drive cell-type-specific expression in brain, including in neuronal progenitors, astrocytes, and oligodendrocytes. MiniPromoters are promoters of less than 4 kb, based on cell-type-specific gene expression in human brain, and verified as faithful and specific in the mouse brain (Portales-Casamar et al., 2010).

Variable penetrance of some genetic mutations, the unexpected behavior of gene-targeted loci, and genetic compensation can confound gene analyses. In mice, genetic background can alter phenotype penetrance, but even in a defined genetic background penetrance can be variable (Dickinson et al., 2016). Additionally, genetic “knockouts” generated by using different strategies (deletion of start exons, all exons, or CRISPR/Cas9 deletion of a few exons) can yield different phenotypes (Hosur et al., 2020). CRISPR/Cas9-mediated gene editing can lead to exon skipping and hypomorphism, neomorphism, or gain of function of the targeted gene, leading to unexpected phenotypes that do not reflect loss of function (Anderson et al., 2017; Chen et al., 2018; Uddin et al., 2015). Phenotypic differences between knockdown and knockout models, including mice, zebrafish, and *Arabidopsis*, could be explained by genetic compensation, by which related genes are upregulated in response to gene knockout but not knockdown (El-Brolosy and Stainier, 2017). Therefore, the development of widespread and long-lasting short hairpin RNA (shRNA) knockdown in mouse embryonic nervous system would be beneficial.

Mutations in *SPTBN2* (encoding the membrane scaffold protein β-III SPECTRIN) are associated with Spinocerebellar Ataxia Type 5 (SCA5) (Cho and Fogel, 2013; Ikeda et al., 2006; Nicita et al., 2019; Ranum et al., 1994; Wang et al., 2014), Spinocerebellar Ataxia Autosomal Recessive 14 (SCAR14) (Elsayed et al., 2014; Lise et al., 2012; Yıldız Bölükbaşı et al., 2017), and ataxic cerebral palsy (Parolin Schnekenberg et al., 2015). In mice, *Sptbn2* targeting recapitulates adult-onset progressive ataxia, but expression of shorter SPTBN2 isoforms (Stankewich et al., 2010) or expression of alternative splice variants (Perkins et al., 2010) suggest that ataxia might reflect hypomorphism or neomorphism rather than complete loss of function.

In this study, we developed a method to manipulate gene expression in the developing murine nervous system termed NEural Plate Targeting by *in Utero* NanoinjEction (NEPTUNE). *In utero* transduction of the neural plate at E7.5 achieved near 100% targeting of the future brain and ca. 80% of the spinal cord. To achieve conditional expression while avoiding the use of Cre mice, we used MiniPromoters (Portales-Casamar et al., 2010) to drive cell-type-specific expression in neuronal progenitors as well as glia. As proof of principle, we knocked down *Olig2*, which recapitulated the phenotypes of the published knockout (Takebayashi et al., 2002). Finally, using NEPTUNE, we knocked down *Sptbn2* and discovered early functions for *Sptbn2* in neural tube development, embryonic turning, and abdominal wall closure. Our results show that NEPTUNE is a powerful method for manipulation of gene expression in the developing nervous system that can reveal functions for human disease-associated genes.

## Results

### Optimal parameters for maximum survival and transduction lead to widespread and stable targeting of developing brain

**Figure 1:**
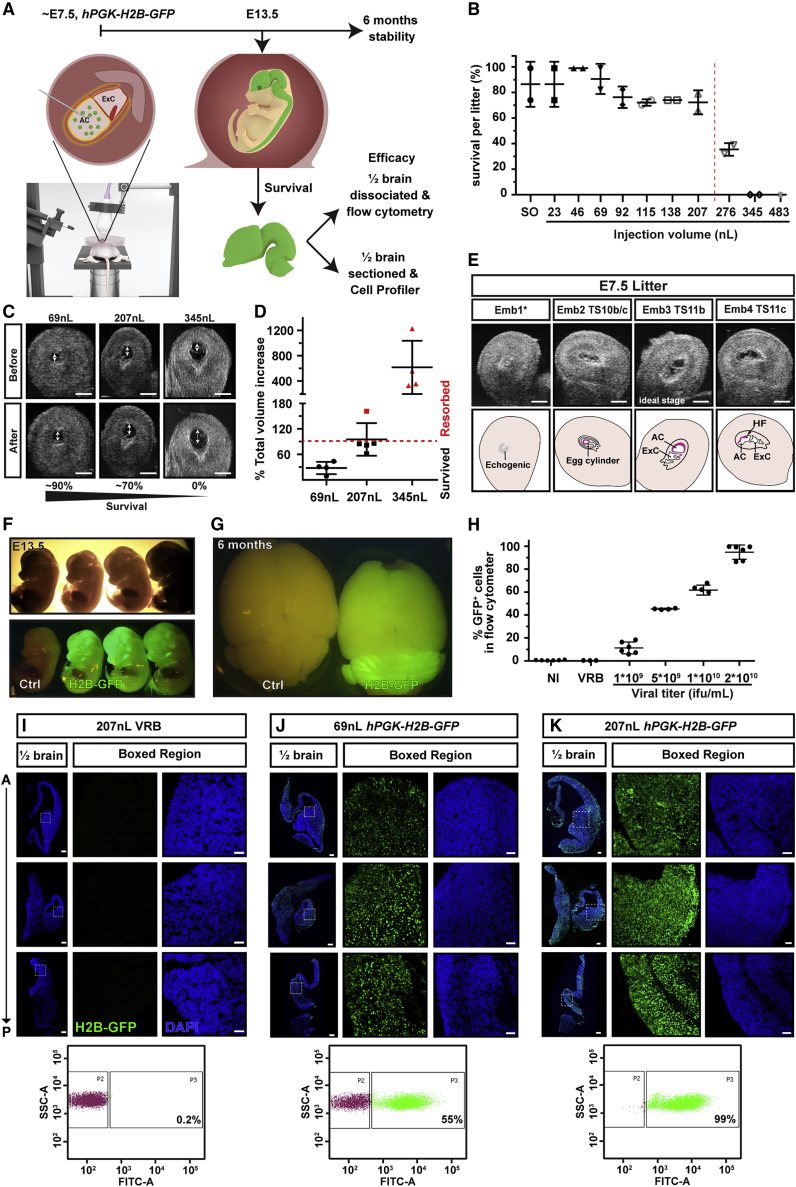
Ultrasound-guided *in utero* nanoinjection of lentivirus at E7.5 results in widespread and stable transduction of the murine CNS (A) The amniotic cavity was injected at E7.5 and embryos were collected at E13.5 or as adults at 6 months. E13.5 brains were divided in half and assessed for GFP positivity by using flow cytometry of dissociated cells or CellProfiler analysis of sectioned brain halves. (B–D) (B) Optimal injection volume was assessed by injecting 23–483 nL of viral resuspension buffer (VRB) at E7.5. Red dotted line denotes drop in survival at volumes >207 nL. Each data point represents survival percentage in one set/litter of injected embryos; for litter details see Figure S1C. SO, surgery only. (C) Distension of the amniotic cavity (double-headed arrows) is visible on ultrasound (scale bars represent 1 mm) and calculating volume after injection showed that (D) survival drops if the total volume increases more than 90%. Each datapoint represents the percent total volume increase in one embryo/amniotic cavity. Red datapoints reflect embryos that were aborted. (E) Ultrasound images of externalized uterus and four embryos (Emb) at E7.5. Theiler stage (TS) 11b was most suitable for high efficiency and high survival. Emb 1∗ is likely resorbing or severely underdeveloped. Scale bars represent 1 mm. (F and G) (F) GFP^+^ heads in whole embryos at E13.5 and (G) GFP signal in brain at 6 months. (H) Targeting efficacy is dependent on viral titer. injections of lentivirus (207 nL) with four titers show dose-dependent transduction, with a titer of 2 × 10^10^ ifu/mL resulting in transduction of 85%–99% of cells in brain at E13.5, assessed by flow cytometry. Each datapoint represents flow-cytometry quantification from one half brain; the other half was used for quantification with CellProfiler. (I and K) Representative images of E13.5 half brains from embryos transduced at E7.5 with VRB, 69 or 207 nL of 2 × 10^10^ ifu/mL hPGK-H2B-GFP virus, with corresponding flow-cytometry data below (n = 3 for each condition). Top row is forebrain, middle row is diencephalon, and bottom row is hindbrain. Scale bars represent 200 μm (half brain) and 50 μm (boxed regions). AC, amniotic cavity; HF, head fold; NI, noninjected; ifu, infectious units.

Prior to neural tube closure in mice, around E7.5, the neural plate is exposed to the amniotic fluid and should be accessible to injected virus, allowing targeting of the future nervous system (Figure 1A). Neurulation stages can be discerned by ultrasound until neural fold fusion of brain at E9.5 (Figure S1A). Injections at E8.5 were too late to achieve consistently high levels of transduction, presumably because neural fold fusion is ongoing (Figure S2). We therefore focused on E7.5 for *in utero* transduction, before neural fold fusion, when the amniotic cavity is discernible by ultrasound and the microcapillary needle tip can be accommodated in the cavity (Figures S1A and S1B). To determine the optimal parameters for embryo survival and maximum transduction, we injected a range of volumes of viral resuspension buffer into the amniotic cavity (23–483 nL) at E7.5 and recorded the percentage of injected embryos per litter surviving at E13.5 (Figures 1B and S1C). Sham surgery identified a baseline resorption rate of 0%–25%. Therefore, we drew the cutoff for acceptable survival rates at 70% and identified 207 nL as the highest permissible volume for normal survival (Figure 1B). At E7.5, embryonic development and amniotic cavity size varies even within litters. Therefore, the best predictor of survival was not volume but the relative increase in amniotic volume, whereby an increase >90% resulted in embryo resorption (Figures 1C and 1D). We therefore selected 207 nL (62%–162% volume increase, mean = 95% ± 38% SD) and 69 nL (9%–44% volume increase, mean = 28% ± 14% SD) for further study. A volume of 345 nL consistently resulted in resorption (325%–1,227% volume increase, mean = 615% ± 421% SD) and was excluded from further experiments. We compared appearance on ultrasound with the Theiler stage (TS) of embryos dissected out immediately after imaging and identified TS 11b as the optimal injection stage for 207 nL. This late neural plate stage, characterized by an elongated allantoic bud, precedes the appearance of the cranial neural (head) folds (HF), which arise at TS 11c (Figure 1E). To optimize selection of females for surgery and take into account the 3Rs (replacement, reduction, refinement), ultrasound confirmation of pregnancy before surgery can be used for staging to ensure ideal conditions (Figure S1D).

After volume optimization for maximum survival, we next determined the viral titers needed to successfully transduce the entire neural plate, using a lentiviral construct that induces stable expression of H2B-GFP (Beronja et al., 2010) (*hPGK-H2B-GFP*). We injected 207 nL of virus at E7.5 and collected embryos at E13.5 or brains at 6 months. Half of each brain at E13.5 was dissociated and analyzed by using flow cytometry, and the other half was sectioned and stained for GFP and DAPI (4′,6-diamidino-2-phenylindole). H2B-GFP^+^ nuclei were quantified by using CellProfiler (McQuin et al., 2018) (Figure 1A). This approach achieved widespread positivity, concentrated in the brain/head region at E13.5 (Figure 1F), which was stable for at least 6 months (Figure 1G). Flow-cytometry and CellProfiler quantification yielded highly correlated quantification of GFP^+^ cells (r^2^ = 0.9945, p < 0.0001, Figure S1E); CellProfiler typically identified 80% of GFP^+^ cells compared with flow cytometry. Different viral titers resulted in dose-dependent transduction of brain between 5.7% and 99.7%, quantified by flow cytometry (Figures 1H: 1 × 10^9^ infectious units per milliliter [ifu/mL], mean = 11.4% ± 5.1% SD; 5 × 10^9^ifu/mL, mean = 45.4% ± 0.5% SD; 1 × 10^10^ ifu/mL, mean = 63.8% ± 3.4% SD; 2 × 10^10^ ifu/mL, mean = 94.8% ± 6.3% SD). However, higher viral titers had a negative impact on embryo survival (Figure S1F). To assess the impact of virus storage on NEPTUNE efficacy, we stored the lentivirus for 3 months at −80°C. Virus storage resulted in mild loss of infectivity *in vitro* (Figure S1G) but a 40% loss of infectivity *in vivo* (Figure S1H, from 94.8% to 53.8%—note that “fresh virus” embryos are the same as in Figure 1H, 2 × 10^10^ ifu/mL), accompanied by ca. 30% improved survival per injected litter (Figure S1I). In conclusion, using the optimal volume (207 nL) and titer (2 × 10^10^ ifu/mL), 95% of cells in the developing brain could reproducibly be targeted with NEPTUNE, confirmed by immunohistochemistry, CellProfiler quantification, and flow cytometry (Figures 1H–1K).

### Lentiviral transduction is even across CNS regions and cell types

The neural tube closes at specific neural tube closure points (Nikolopoulou et al., 2017), making these regions potentially less amenable to lentiviral manipulation due to decreased accessibility and subsequent uneven viral transduction. In mice, the initial neural tube closure point 1 is at the hindbrain/cervical boundary, closure point 2 is at the forebrain/midbrain boundary, and closure point 3 is at the most rostral end of the forebrain. Closure/zippering proceeds rostrally and caudally from closure points 1 and 2, whereas closure proceeds caudally from closure point 3 toward closure point 2 (Nikolopoulou et al., 2017). Injections at E8.25–E8.5 (Figure S2A) reflected partial neural tube closure with variable efficiency of transduction of forebrain, midbrain, hindbrain, and spinal cord at E11.5 (Figures S2B–S2E). Transduction was highest in forebrain, the region which is open longest (mean = 24% ± 11.1% SD) and lowest in hindbrain (mean = 5.5% ± 2.7% SD), the region which closes first (Figures S2B and S2C). Transduction was around 8% in thoracic and lumbar spinal cord (thoracic mean = 6.2% ± 3.8% SD; lumbar mean = 10.3% ± 6.4% SD) (Figures S2D and S2E).

**Figure 2:**
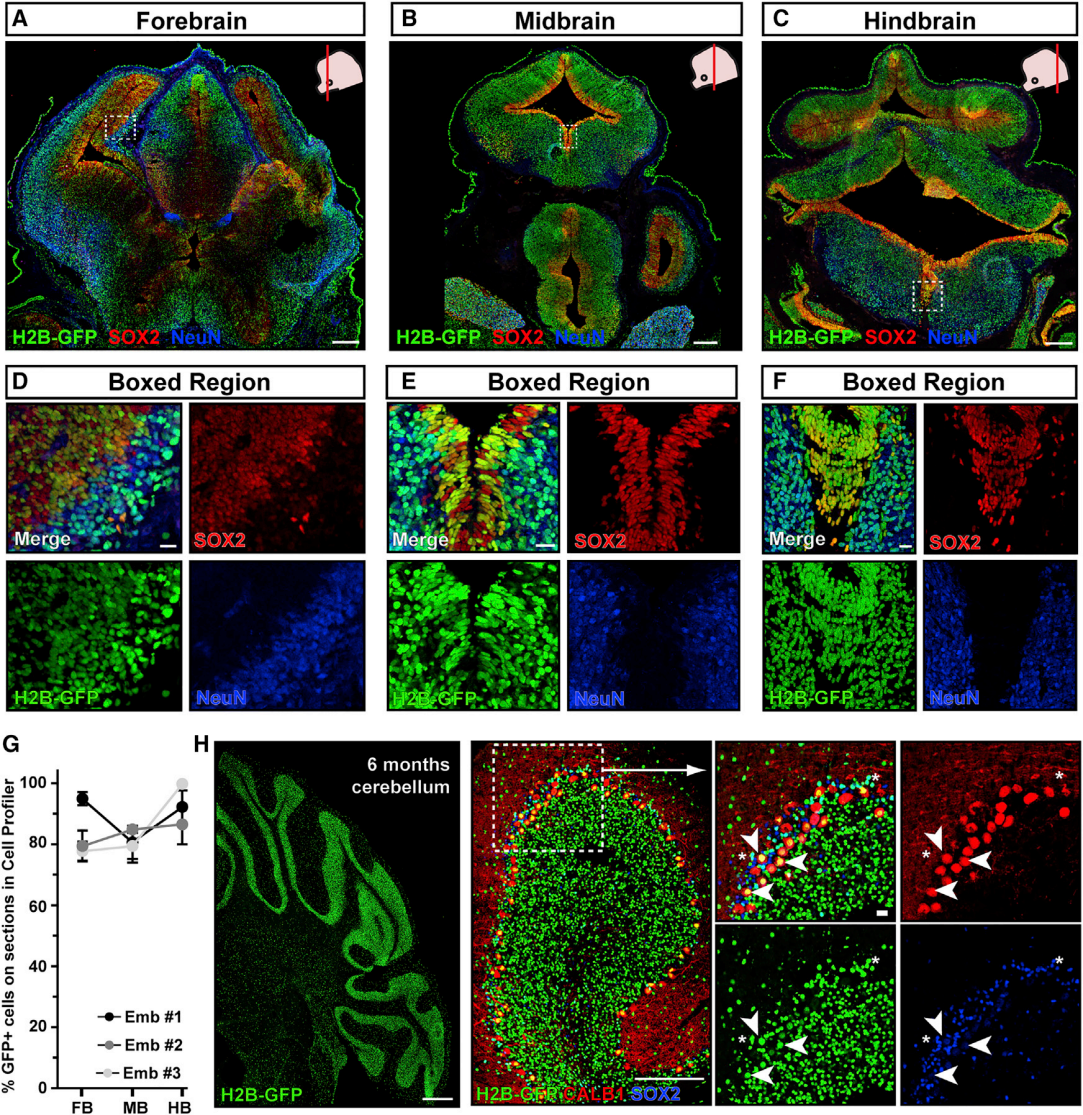
E7.5 NEPTUNE results in even transduction across brain and neural cell types (A–F) GFP transduction efficiency in (A) forebrain, (B) midbrain, and (C) hindbrain of E13.5 brain, transduced at E7.5 with amniotic cavity injection, stained for GFP, neural progenitors (SOX2), and neurons (NeuN), and magnification of H2B-GFP expression in both (D–F) progenitors and differentiating cells (representative images of n = 3). Scale bars represent 200 μm (A–C) and 20 μm (D–F). (G) Quantification of GFP^+^ cells in forebrain (FB), midbrain (MB), and hindbrain (HB) using CellProfiler. (H) Transduction at E7.5 also contributes to cerebellum, labeling both neurons (CALB1, indicated by white arrowheads) and glia (SOX2, indicated by white asterisks) at 6 months (representative of n = 4). Scale bars represent 200 μm in overviews (left two panels) and 20 μm in boxed regions.

**Figure 3:**
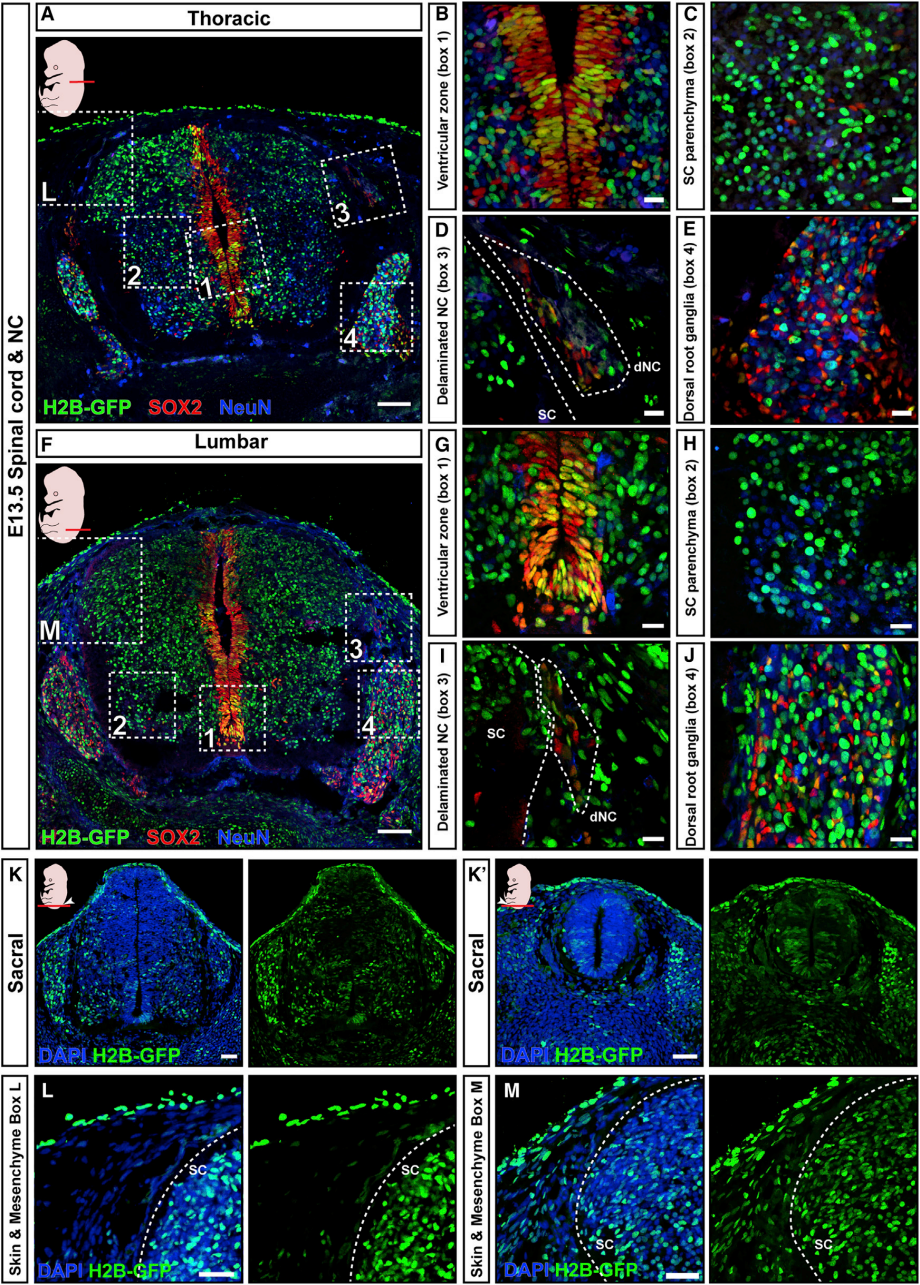
E7.5 NEPTUNE results in even transduction across spinal cord and neural cell types (A–J) GFP transduction of cells contributing to (A–E) thoracic and (F–J) lumbar E13.5 spinal cord (SC), by E7.5 amniotic cavity injection. Spinal cord stained for GFP, neural progenitors (SOX2), and neurons (NeuN). H2B-GFP^+^ neural progenitors in the ventricular zones (B and G), H2B-GFP^+^/NeuN^+^ neurons in spinal cord parenchyma (C and H), H2B-GFP^+^ delaminating neural crest cells (dNC, D and I), and H2B-GFP^+^ dorsal root ganglia (E and J). (K and K′) H2B-GFP^+^ cells in sacral spinal cord. (L) H2B-GFP^+^ cells in spinal cord and skin, but not mesenchyme, in thoracic levels. (M) H2B-GFP^+^ cells in mesenchyme, spinal cord, and skin, at lumbar levels (representative images for n = 3). Scale bars represent 200 μm in (A) and (F), 10 μm in (B)–(E) and (G)–(J), and 50 μm in (K), (K′), (L), and (M).

We therefore focused on E7.5 injections and assessed transduction efficiency throughout the brain and spinal cord, in neural precursors or neurons, to determine whether the entire nervous system was evenly targeted by injection at this stage (Figures 2 and 3, split channels for Figures 3B–3E and 3G–3J in Figure S3). H2B-GFP was highly and evenly expressed from forebrain to hindbrain, in both SOX2^+^ neural precursors and post-mitotic NeuN^+^ neurons (Figures 2A–2G). H2B-GFP was also widely expressed in the cerebellum at 6 months (Figure 2H), which develops from a cerebellar primordium first evident at E12.5. H2B-GFP was stably expressed in both GFP^+^ calbindin-positive (CALB1^+^) Purkinje neurons and SOX2^+^ glia (Figure 2H).

A similarly even transduction pattern was present in the spinal cord at thoracic levels, which form via primary neurulation (Figures 3 and S3). Virus injection at E7.5 efficiently transduced the neural plate contributing to SOX2^+^ progenitor cells and NeuN^+^ mature neurons in the spinal cord ventricular zone and parenchyma (Figures 3A–3C), as well as delaminating neural crest cells (Figures 3A and 3D) and dorsal root ganglia (Figures 3A and 3E). Spinal cord transduction efficiency was slightly lower than the corresponding brain transduction (Figure S3B, brain control data from brain halves analyzed by CellProfiler and also presented in Figure S1E, black data points).

Lumbar and sacral spinal cord is formed via secondary neurulation from the caudolateral epiblast, which contains neuromesodermal progenitors (NMPs) that are internalized around E8.5–E9.0 (Wymeersch et al., 2021). *In utero* nanoinjection at E7.5 results in widespread H2B-GFP positivity in caudal embryo in lumbar and sacral levels (Figures 3F–3K); there was H2B-GFP positivity not only in skin and spinal cord but also in mesenchyme, suggesting targeting of NMPs contributing to both neural and mesodermal tissues (Figures 3L and 3M—note the H2B-GFP positivity in mesenchyme in Figure 3M, compared with absence of H2B-GFP in mesenchyme in head or thoracic spinal cord sections in Figures 3L and 2A–2C). In conclusion, *in utero* nanoinjection at E7.5 targets brain and spinal cord formed by primary or secondary neurulation.

### Cell-type-specific expression with NEPTUNE and MiniPromoters

**Figure 4:**
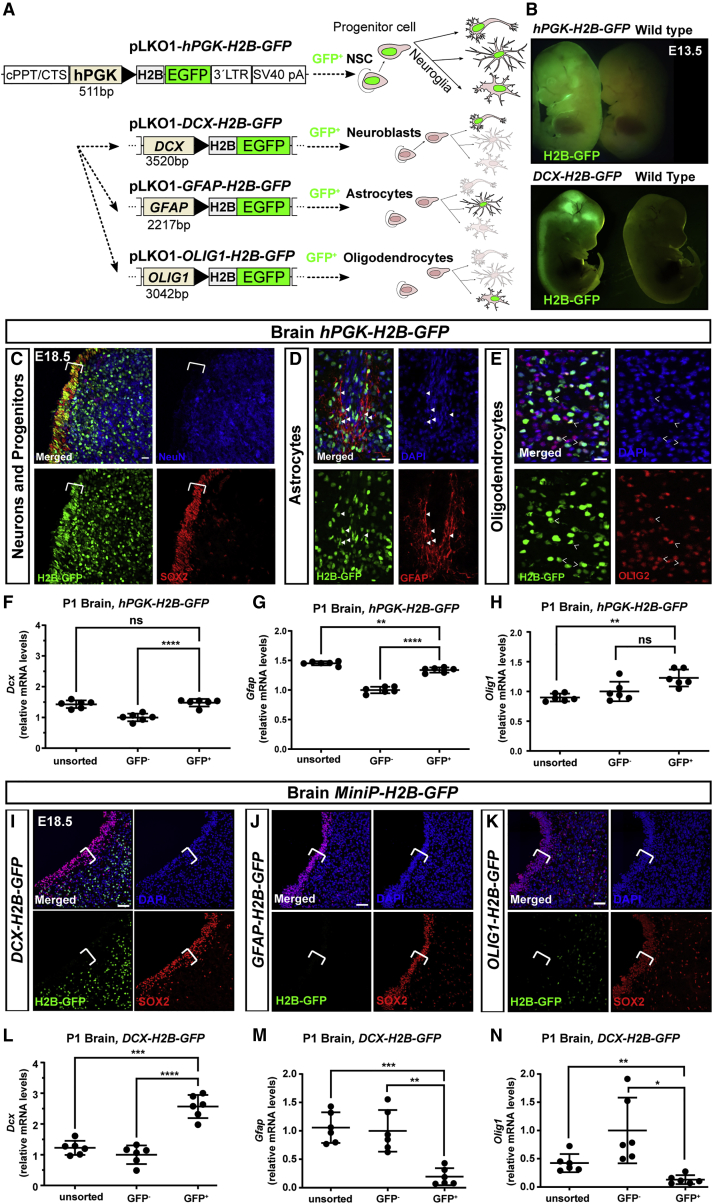
Cell-type-specific expression with NEPTUNE and MiniPromoters (A) Cloning schematic: the *hPKG* promoter in *hPGK-H2B-GFP* was replaced with MiniPromoters for *DCX*, *OLIG1*, or *GFAP* for expression in neuronal progenitors, astrocytes, or oligodendrocytes. (B–N) Amniotic cavities were injected at E7.5 and embryos were collected for analysis at (B) E13.5, (C–E and I–K) E18.5, or (F–H and L–N) P1. (B) Uninjected littermate (Ctrl) and an embryo injected with *hPGK-H2B-GFP*, with GFP^+^ expression in skin as well as brain. GFP signal in a *Dcx-H2B-GFP* transduced embryo is restricted to the brain and spinal cord, with no obvious GFP in skin. (C–E) At E18.5, *hPGK-H2B-GFP* is expressed in neural progenitors (SOX2^+^) and neurons (NeuN^+^), here shown in ventrolateral midbrain (C). Bracket in (C) denotes proliferating SOX2^+^ ventricular zone precursors. (D) At E18.5, *hPGK-H2B-GFP* is expressed in astrocytes (GFAP^+^), shown here in the indusium griseum; arrowheads denote GFP^+^ GFAP^+^ cells. (E) At E18.5, *hPGK-H2B-GFP* is expressed in oligodendrocytes (OLIG2^+^), shown here in cerebellum. Open arrowheads denote GFP^+^ OLIG2^+^ cells. Scale bars are in (C) to (E) represent 10 μm (representative images of n = 6). (F–H) qPCR analysis for enrichment of (F) neurons (*Dcx*), (G) astrocytes (*Gfap*), and (H) oligodendrocytes (*Olig1*) in P1 brains, injected with *hPGK-H2B-GFP* at E7.5. (I–K) GFP expression at E18.5, from (I) *DCX-H2B-GFP*, (J) *GFAP-H2B-GFP*, or (K) *OLIG1-H2B-GFP* viruses injected at E7.5. None of the constructs was expressed in SOX2^+^ neural stem cells (white brackets). (I) H2B-GFP expression in post-mitotic parenchyma in *DCX-H2B-GFP-*injected embryos (representative of n = 6), shown here in ventrolateral midbrain. H2B-GFP expression is generally mutually exclusive with SOX2 (one single double-positive cell in this section). (J) Little to no expression of H2B-GFP in parenchyma of *GFAP-H2B-GFP*-injected embryos (representative of n = 4), shown here in ventrolateral midbrain. (K) Scattered H2B-GFP^+^ cells in brain parenchyma of *OLIG1-H2B-GFP*-injected embryos in both SOX2^+^ and SOX2^−^ cells, shown here in ventrolateral midbrain (representative of n = 4). (L–N) qPCR analysis for enrichment of (L) neurons (*Dcx*), (M) astrocytes (*Gfap*), and (N) oligodendrocytes (*Olig1*) in P1 brains, injected with *DCX-H2B-GFP* at E7.5. qPCR: all values are normalized to *Actb*. Each dot represents one brain (n = 6 per condition). Differences in expression levels were analyzed with one-way ANOVA and Dunnett's multiple comparisons test. ns, not significant; ∗∗p < 0.01, ∗∗∗p < 0.001, ∗∗∗∗p < 0.0001. Scale bars represent 20 μm in (C)–(E) and 50 μm in (I)–(K).

NEPTUNE targets the entire central nervous system, as well as the neural crest and NMPs contributing to caudal mesoderm (Figures 1, 2, and 3). To achieve a conditional method to investigate gene function in brain, we next aimed to develop cell-type-specific expression and bypass dependence on Cre mouse strain availability. We cloned MiniPromoter sequences identified by the Pleiades Promoter Project (Portales-Casamar et al., 2010) as driving expression in neuronal progenitors (*Doublecortin*, *DCX* miniP), astrocytes (*Glial Fibrillary Acidic Protein*, *GFAP* miniP), and oligodendrocytes (*Oligodendrocyte Transcription Factor 1*, *OLIG1* miniP), and replaced the *hPGK* promoter driving *H2B-GFP* in *hPGK-H2B-GFP* (also known as *LV-GFP*), creating instead *DCX-H2B-GFP*, *GFAP-H2B-GFP*, and *OLIG1*-*H2B-GFP* (Figure 4A). *In utero* transduction with the parental *hPGK-H2B-GFP* at E7.5 led to widespread expression in skin (Figure 4B, top; also evident in Figures 2A–2C, 3A, 3F, 3K, and 3K′) as well as in the nervous system in SOX2^+^ neural progenitors (Figure 4C), NeuN^+^ neurons, GFAP^+^ astrocytes (Figure 4D), and OLIG2^+^ oligodendrocytes (Figure 4E). qPCR of sorted H2B-GFP^+^ cells from P1 brain, injected at E7.5 with *hPGK-H2B-GFP*, showed that GFP^+^ brain cells were 1.48-fold enriched for *Dcx* (±0.12 SD, Figure 4F, and 1.34-fold enriched for *Gfap* (±0.04 SD, Figure 4G) compared with GFP^−^ cells, but not significantly enriched for *Olig1* (1.23-fold, ±0.14 SD, Figure 4H), suggesting generally similar targeting of neural plate contributing to neuroblasts, astrocytes, and oligodendrocytes.

In contrast, transduction with *DCX-H2B-GFP* resulted in specific nervous system GFP expression with clear signal in brain and spinal cord (Figure 4B, bottom). GFP expression in *DCX-H2B-GFP* embryos was excluded from SOX2^+^ progenitors (Figure 4I) and was instead expressed in DCX^+^ parenchyma (Figure S4A). *GFAP-H2B-GFP* and *OLIG1*-*H2B-GFP* could not be detected in whole embryos (data not shown), and expression of H2B-GFP was excluded from SOX2^+^ progenitors for both (Figures 4J and 4K). In *GFAP-H2B-GFP* embryos, GFP^+^ nuclei were specifically present in GFAP^+^ cells (Figure S4B, indusium griseum region, the same region as in Figure 4D). In *OLIG1-H2B-GFP* embryos, GFP^+^ nuclei were widespread in parenchyma (Figure 4K) and expression was not exclusive to OLIG1^+^ cells (Figure S4C). qPCR of GFP^+^ and GFP^−^ cells from P1 brain injected at E7.5 with *DCX-H2B-GFP* showed that *DCX-GFP*^+^ brain cells were 2.6-fold enriched for *Dcx* (±0.38 SD, Figure 4L) but depleted for *Gfap* (0.19-fold expression ± 0.15 SD, Figure 4M) and depleted for *Olig1* (0.13-fold expression ± 0.08 SD, Figure 4N). Thus, NEPTUNE generally results in expression in neuroblasts, astrocytes, and oligodendrocytes, whereas NEPTUNE with *DCX-H2B-GFP* results in cell-type-specific expression in neuroblasts/neurons. *GFAP-H2B-GFP* is specifically expressed in astrocytes whereas *OLIG1-H2B-GFP* is more promiscuous.

**Figure 5:**
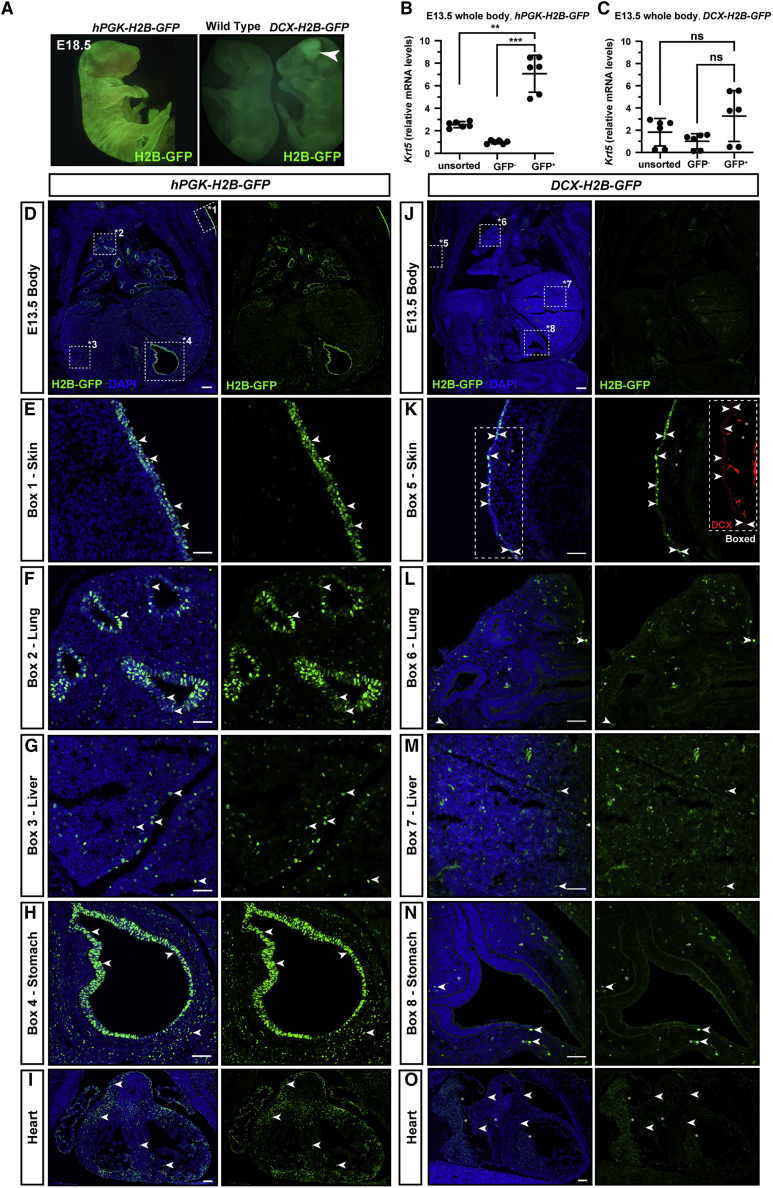
*DCX-H2B-GFP* drives specific expression in neurons (A) NEPTUNE with DCX-H2B-GFP, embryos collected at E18.5. GFP expression in an *hPGK-H2B-GFP* embryo in skin. GFP expression in a *DCX-H2B-GFP* embryo in brain (arrowhead) but not skin. (B and C) qPCR for skin marker *Keratin 5* (*Krt5*) in (B) *hPGK-H2B-GFP* embryos and (C) *DCX-H2B-GFP* embryos at E13.5. (D–I) Embryos (amniotic cavities) were injected with *hPGK-H2B-GFP* virus at E7.5, collected at E13.5, and sectioned and stained for GFP and DAPI. (D) Tile-scan overview of internal organs, with boxes denoting panels in (E) to (H) (representative of n = 3). GFP^+^ cells in (E) skin, (F) lung epithelia, (G) liver, (H) stomach epithelium, and (I) heart. (J–O) Embryos (amniotic cavities) were injected with *DCX-H2B-GFP* virus at E7.5, collected at E13.5, and sectioned and stained for GFP and DAPI (representative of n = 3). (J) Tile-scan overview of internal organs, with boxes denoting panels in (K) to (N). (K) GFP^+^ cells in skin, overlapping with low-level expression of DCX (in red, inset). GFP^+^ cells in lung (L), liver (M), stomach (N), and heart (O). In all, arrowheads denote GFP signal/cells. qPCR: *Krt5* levels are normalized to *Actb*. Each dot represents one embryo (n = 6 per condition). Differences in expression levels were analyzed with one-way ANOVA and Dunnett’s multiple comparisons test. ns = not significant, ∗∗p < 0.01, ∗∗∗p < 0.001. Scalebars in (D) and (J), 200 mm. Scalebars in (E)–(H) and (K)–(N), 50 mm. Scalebars in (I) and (O), 100 mm.

To comprehensively assess which cells are transduced at E7.5 and express the *DCX-H2B-GFP* construct, we compared expression of H2B-GFP in *hPGK-H2B-GFP-* and *DCX-H2B-GFP-*injected whole embryos. At E18.5, GFP expression was prominent in skin of *hPGK-H2B-GFP-*injected embryos (Figure 5A, left) whereas GFP expression in *DCX-H2B-GFP-*injected embryos was most prominent in cortex (Figure 5A, right), and skin positivity was comparable with an uninjected control. qPCR of GFP^+^ cells sorted from whole bodies at E13.5 confirmed 7.1-fold enrichment of skin targeting by *hPGK-H2B-GFP* (±1.3 SD, Figure 5B) but no targeting by *DCX-H2B-GFP* (Figure 5C). In *hPGK-H2B-GFP-*injected embryos, the GFP^+^ signal was clear and abundant in epithelial cells in skin, lung, and stomach, and scattered GFP^+^ cells could be found in the liver and heart (Figures 5D–5I). qPCR analysis of sorted GFP^+^ cells from E13.5 *hPGK-H2B-GFP* whole bodies (Figure S5) further confirmed widespread transduction predominantly in cells either in contact with amniotic fluid or which are expected to be derived from the neural crest, including cells expressing *Dcx* (neuroblasts, Figures S5A and S5B), *Sox2* (neural stem cells and epithelial cells, for example in the lung [Que et al., 2009], Figure S5D), and *Cdh1* (also known as epithelial cadherin, in epithelial cells, Figure S5E). Similar to E18.5 brain analysis, *hPGK-H2B-GFP* GFP^+^ cells at E13.5 were not significantly enriched for *Olig1* (Figure S5C) and were depleted for *CD31/Pecam-1* and *CD34* (markers of vascular and hematopoietic cells, Figures S5F and 5G). In sum, these data suggest that NEPTUNE preferentially targets epithelial cells and neural plate.

In contrast, *DCX-H2B-GFP-*injected embryos confirmed 5.1-fold enrichment of *Dcx* in GFP^+^ sorted cells from whole-body analysis by qPCR at E13.5 (Figure S5B, right panel) compared with 2.5-fold enrichment with *hPGK-H2B-GFP* (Figure S5B, left panel). Similar to results for brain at E18.5 (Figure 4N), *Olig1* was depleted in *DCX-*GFP^+^ cells (Figure S5C). *Sox2* was less enriched in *DCX*-GFP^+^ cells than in *hPGK*-GFP cells (1.4-fold versus 2.2-fold for *Sox2*, Figure S5D). *Cdh1* enrichment was similar in both conditions (3.3-fold in *hPGK*-GFP^+^ cells and 3-fold in *DCX-GFP*^+^ cells, Figure S5E). Similar to *hPGK-H2B-GFP*, *DCX-H2B-GFP* does not appear to transduce or be expressed in cells expressing *CD31* or *CD34* (Figures S5F and S5G). In *DCX-H2B-GFP* whole-embryo sections at E13.5 (Figure 5J) GFP could be detected in some skin cells, which could reflect low-level expression of DCX in skin (Figure 5K). GFP was not detected, or expressed in only a few scattered cells, in parenchyma (not epithelia) in the lungs, liver, stomach, and heart (Figures 5L–5O).

In conclusion, NEPTUNE achieves >95% transduction of brain with the *hPGK* promoter, and expression can be directed to specific cell types by using MiniPromoters, with an efficiency dependent on the promoter used.

### NEPTUNE knockdown of *Olig2* recapitulates knockout phenotypes

**Figure 6:**
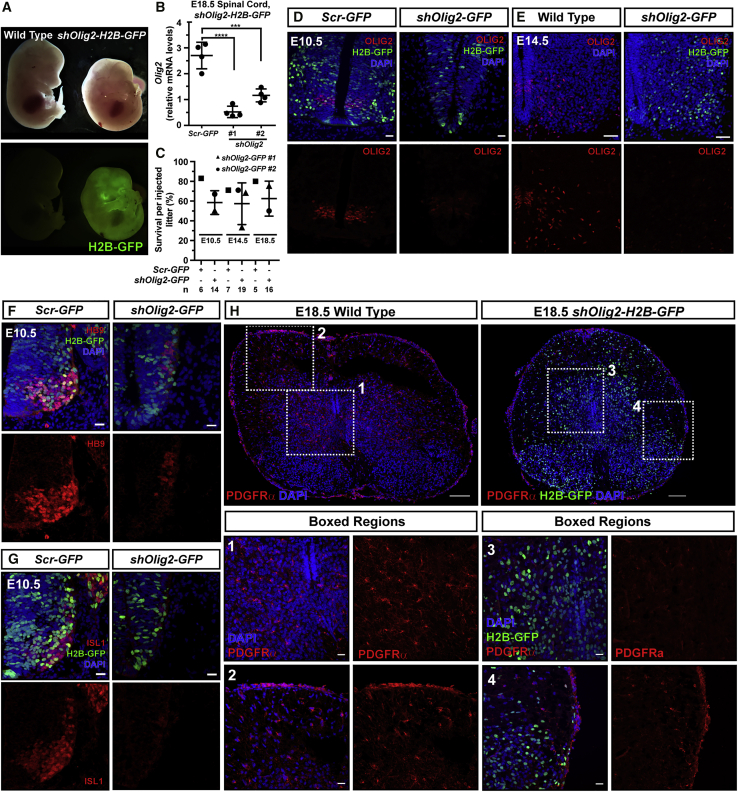
NEPTUNE-mediated shRNA knockdown of *Olig2* recapitulates knockout phenotypes *in vivo* NEPTUNE with virus encoding *shOlig2-H2B-GFP*, or *Scr-H2B-GFP*, and embryos collected at E10.5, E14.5, or E18.5. (A) Shorter crown-rump length of E14.5 *shOlig2-H2B-GFP* embryos. (B) qPCR for *Olig2* in spinal cord at E18.5, from *Scr-H2B-GFP-* and *shOlig2-H2B-GFP*-injected embryos. Each dot represents spinal cord from one embryo (n = 4 per condition). In the following panels, both shRNAs yielded equivalent phenotypes. (C) Survival of embryos injected with *shOlig2-H2B-GFP* at E10.5, E14.5, and E18.5. Both shRNAs were tested. Average survival was 60% at all three stages. Each dot represents the percentage survival of injected embryos per litter; n (embryos injected) is specified per condition under the table. (D–E) OLIG2 expression in pMN of (D) E10.5 and (E) E14.5 spinal cord in control (*Scr-H2B-GFP* or uninjected) embryos and *shOlig2-H2B-GFP* embryos. (F) HB9^+^ and (G) Islet1^+^ motoneurons in the MN (motoneuron) domain in E10.5 spinal cord in control embryos. Reduced size of ventral horn and MN domain in *shOlig2-H2B-GFP* spinal cord. HB9^+^ or Islet1^+^ cells dorsal to MN domain present in *shOlig2-H2B-GFP* embryos. (H) PDGFRα (red) in spinal cord at E18.5. In controls, PDGFRα is present in spinal cord oligodendrocytes (Boxed region 1) and meningeal vasculature (Boxed region 2). In *shOlig2-H2B-GFP* spinal cord, PDGFRα^+^ oligodendrocytes are absent (Boxed region 3), whereas PDGFRα is present in meningeal vasculature (Boxed region 4) (representative images of n = 3 for Scr-GFP or uninjected and n = 6 for *shOlig2-H2B-GFP* condition for E10.5, E14.5, and E18.5). qPCR: *Olig2* levels are normalized to *Actb*. Differences in expression levels were analyzed with one-way ANOVA and Dunnett's multiple comparisons test. ∗∗∗p < 0.001, ∗∗∗∗p < 0.0001. Scale bars represent 20 μm in (D), (F), and (G) (E10.5) and 50 μm in (E) (E14.5). Scale bars in (H) represent 100 μm (E18.5; top panels) and 20 μm (boxed regions 1–4).

As proof of principle, we next aimed to test whether NEPTUNE could recapitulate established nervous system phenotypes. *Olig2* is a transcription factor required for development of motoneurons and oligodendrocytes, and *Olig2*^−/−^ embryos display a striking phenotype with a shortened crown-rump length in newborn pups and a reduction in HB9/Islet1^+^ motoneurons and platelet-derived growth factor receptor α-positive (PDGFRα^+^) oligodendrocytes (Takebayashi et al., 2002). We expect that crown-rump length shortening is a strong phenotype that can only be recapitulated with robust knockdown throughout the spinal cord. Knockdown of *Olig2*, using either of two different shRNA constructs, with NEPTUNE resulted in a shortened crown-rump length at E14.5 (Figure 6A), and qPCR for *Olig2* in whole spinal cord at E18.5 confirmed RNA silencing by both shRNAs (Figure 6B). Survival of injected embryos was similar to that under control conditions at E10.5, E14.5, and E18.5 (Figure 6C). OLIG2 protein expression in the spinal cord motor neuron progenitor domain (pMN) was nearly absent or absent in *shOlig2-H2B-GFP* embryos at E10.5 and E14.5 (Figures 6D and 6E). *Olig2* knockdown resulted in a decrease in HB9^+^ and Islet1^+^ motoneurons at E10.5 (Figures 6F and 6G) and a loss of PDGFRα^+^ oligodendrocytes at E18.5 (Figure 6H, boxed regions 1 and 3). Importantly, PDGFRα expression was still obvious in vasculature surrounding the spinal cord (Figure 6H, boxed regions 2 and 4). Thus, *Olig2* knockdown with NEPTUNE recapitulated the *Olig2*^*−/−*^ phenotype (Takebayashi et al., 2002).

### NEPTUNE reveals a role for *Spbtn2* in neurulation and abdominal wall closure

Finally, to test whether NEPTUNE can provide insights into gene function or human disease, we focused on a gene associated with neurodevelopmental defects. *Spectrin Beta*, *Non-Erythrocytic 2* (*Sptbn2*) is highly expressed in the CNS and mediates intracellular vesicle transport and cytoskeleton dynamics (Liem, 2016). Mutations in *SPTBN2* are associated with SCA5 (OMIM: 600224) (Cho and Fogel, 2013; Ikeda et al., 2006; Nicita et al., 2019; Ranum et al., 1994; Wang et al., 2014) and SCAR14 (OMIM: 615386) (Elsayed et al., 2014; Lise et al., 2012; Yıldız Bölükbaşı et al., 2017). Mouse models for *Sptbn2* mutation display adult-onset defects in motor function, but these mice express shorter SPTBN2 isoforms (Stankewich et al., 2010) or splice variants (Perkins et al., 2010), suggesting that these models might not reflect complete loss of function. We therefore decided to test whether knockdown of *Sptbn2* at E7.5 induced an ataxic phenotype or a more severe phenotype, as would be expected if the splice variants or shorter variants can compensate for loss of full-length SPTBN2.

Five shRNAs against *Sptbn2* were tested in the neural cell line NE4C, revealing similar 50%–60% downregulation of *Sptbn2* mRNA levels for all constructs, compared with scrambled control, at 24 and 48 h (Figures S6A and S6B). After cloning two of the shRNAs into *hPGK-H2B-GFP* (#1 and #2), knockdown efficacy was maintained (Figures S6C and S6D).

**Figure 7:**
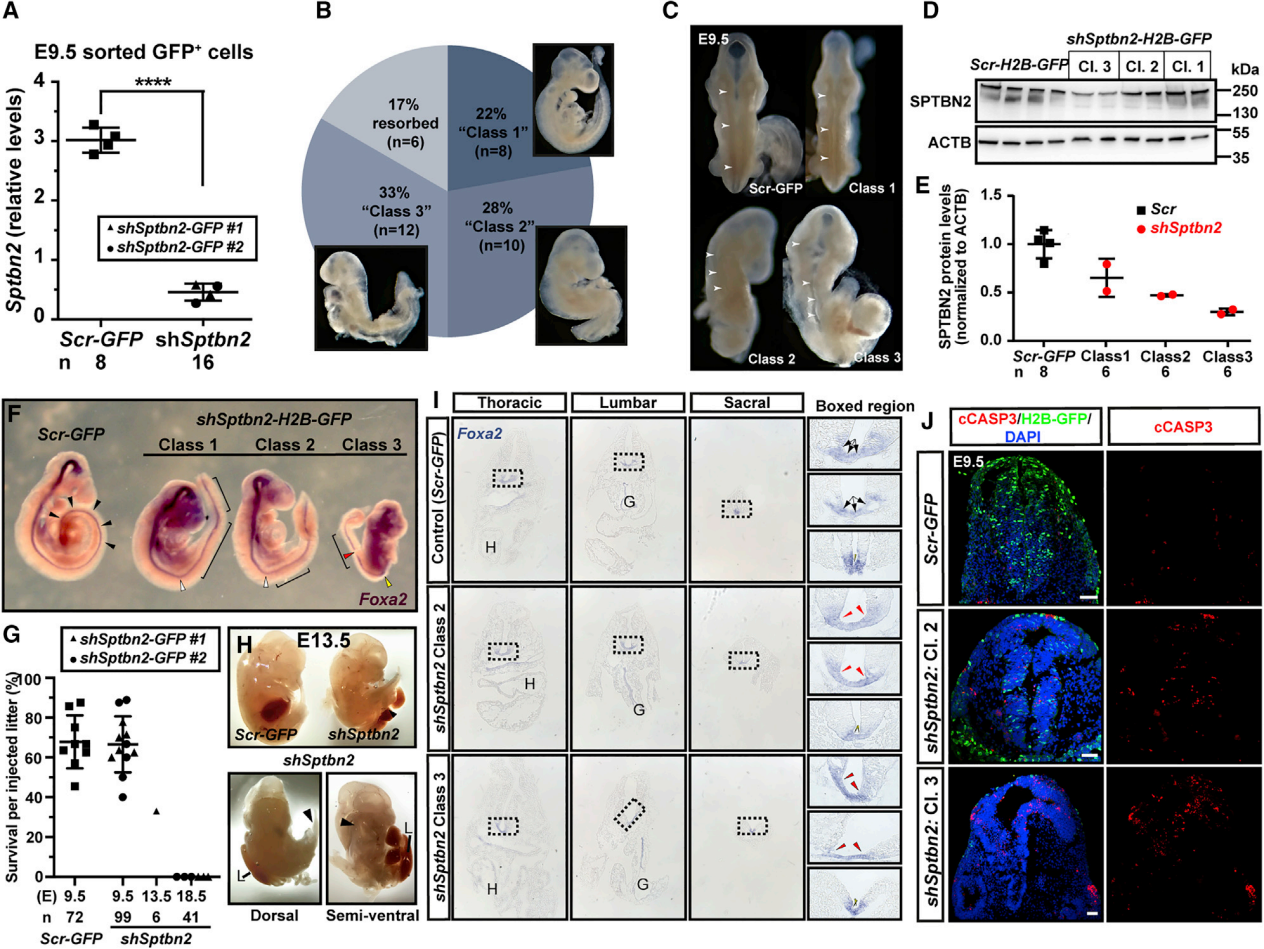
NEPTUNE-mediated shRNA knockdown of *Sptbn2* impairs survival and causes dose-dependent defects in neural tube and in embryonic turning NEPTUNE with virus encoding *shSptbn2-H2B-GFP* or *Scr-H2B-GFP*, and embryos collected at E9.5, E13.5, or E18.5 (A) qPCR for *Sptbn2* in GFP^+^ cells sorted from whole embryos at E9.5. Two *Scr-H2B-GFP* embryos were pooled per “Scr” sample (each dot = 2 embryos). Three to four *shSptbn2-H2B-GFP* embryos were pooled per sample (each dot = 3 or 4 embryos). (B) At E9.5, *shSptbn2-H2B-GFP* embryos presented with three phenotypic severities; 17% are resorbed. Class 1 phenotype (22%) was defined as a straightened body axis, Class 2 phenotype (28%) as a body with a rightward-skewed lumbar and sacral region, and Class 3 phenotype (33%) as developmentally delayed and failure to undergo embryonic turning. (C) Dorsal views of neural tube of control *Scr-H2B-GFP-* and *shSptbn2-H2B-GFP-*injected embryos from the three classes. In control, arrowheads denote a straight spinal cord with even closure. In Class 1–3 *shSptbn2-H2B-GFP* embryos, arrowheads denote kinks in the spinal cord with uneven closure. (D and E) Western blot and quantification of SPTBN2 in whole embryos injected with *Scr-H2B-GFP* or *shSptbn2-H2B-GFP*. Embryos were pooled for analysis, two embryos per *Scr-H2B-GFP* lane and three per *shSptbn2-H2B-GFP* lane. Equal protein amounts were loaded. (D) Western blot for SPTBN2. (E) Quantification of SPTBN2 levels in (D), normalized to ACTB. n (number of embryos included in analysis, contributing to pools) is depicted below each condition. SPTBN2 levels are inversely correlated with phenotype severity. (F) *In situ* hybridization for *FoxA2* in *Scr-H2B-GFP* or *shSptbn2-H2B-GFP* E9.5 embryos. White arrowheads denote an angulated flexure point in Class 1 and Class 2 embryos. Yellow arrowhead denotes the failed turning point in a Class 3 embryo. Red arrowhead denotes an interruption in floorplate Foxa2 in the Class 3 embryo. Brackets denote straightened portions of neural tube. (G) Survival of injected embryos per injected litter in *Scr-H2B-GFP-* or *shSptbn2-H2B-GFP*-injected litters at E9.5, E13.5, and E18.5. Each dot represents the percentage survival in one litter. The total number of injected embryos/amniotic cavities is depicted under the graph. No *shSptbn2-H2B-GFP* embryos survived until E18.5. (H) Dorsal and semi-ventral views of an E13.5 *shSptbn2-H2B-GFP* embryo demonstrates rightward skewing and an abdominal wall closure defect with externalized internal organs including the liver. (I) Sections of embryos in (F) from thoracic to sacral spinal cord. Boxed regions depict a wider floorplate in Class 2 embryos (red arrowheads), and a wider floorplate consisting of a single layer of cells in the lumbar region of Class 3 embryos (red arrowheads in lumbar boxed region). Note the skewed spinal cord floorplate compared with the body axis in Class 3. H, heart; G, gut. (J) Expression of cleaved CASPASE 3 (cCASP3) in E9.5 spinal cord (representative of n = 3 for *Scr-H2B-GFP*; n = 6 for *shSptbn2-H2B-GFP* Class 2 and 3, respectively). Scale bars represent 50 μm. Differences in expression levels in (A) were analyzed with one-way ANOVA and Dunnett's multiple comparisons test, ∗∗∗∗p < 0.0001. Differences in (D) were not tested statistically because two pools per condition should not be statistically tested. Differences in (G) were not tested statistically because only one litter was included at E13.5, due to an obvious impact on survival, and 3R considerations precluded further experiments.

Sorting of *shSptbn2*-GFP^+^ cells from E9.5 whole embryos, injected at E7.5, confirmed silencing of *Sptbn2* mRNA by 80% in targeted cells (Figure 7A). Of 36 embryos injected at E7.5 and analyzed at E9.5, six (17%) were resorbed (similar to baseline resorption rates, Figure 1B), and the remaining 83% could be divided into three classes by phenotype (Figure 7B). We defined Class 1 as the mildest phenotype, with a subtle straightening of the body axis (eight embryos, 22%). Class 2 was characterized by a failure to undergo embryonic turning and a skewed body axis (ten embryos, 28%), and Class 3 was developmentally delayed and had not undergone turning (12 embryos, 33%, Figure 7B). Dorsal views of these three classes revealed a kinked and asymmetric neural tube in all three classes (Figure 7C).

To determine whether *Sptbn2* knockdown resulted in reduced SPTBN2 protein levels, we first sorted GFP^+^ cells from *shSptbn2-H2B-GFP-* and *hPGK-H2B-GFP*-injected embryos for western blotting. However, this yielded highly variable results, which might reflect degradation of SPTBN2 in dissociated cells (Figure S6F—note distinct bands in crushed positive control whole cerebellum versus smearing and extra bands in dissociated cerebellum). We therefore injected embryos with *shSptbn2-H2B-GFP* or *hPGK-H2B-GFP* and collected embryos at E9.5, pooling three embryos per wild-type pool and four embryos by class severity per pool, and adjusted samples for equal total protein concentration. There were reduced levels of SPTBN2 in all three classes, with the greatest reduction in Class 3, the most severely affected embryos (Figures 7D and 7E).

The neural floorplate marker *Foxa2* highlighted the straightened body axis versus failure to undergo turning in Class 1 and Class 3 embryos, respectively, and further highlighted interrupted *Foxa2* signal in spinal cord of Class 3 embryos (Figure 7F). In total, we injected 19 litters of mice with virus encoding *shSptbn2* (construct #1 or #2) at E7.5 and collected embryos at E9.5, E13.5, and E18.5. Surprisingly, no embryos were recovered at E18.5 (6 litters, 3 injected with shRNA #1 and 3 injected with shRNA #2, total 41 embryos, Figure 7G). At E9.5, *shSptbn2-H2B-GFP-*injected embryo survival was similar to that of *hPGK-H2B-GFP-*injected embryo (12 litters, 6 injected with shRNA#1 and 6 injected with shRNA#2, 67 of 99 embryos survived, Figure 7G). At E13.5 only 33% of injected embryos survived (1 litter, 2 of 6 embryos injected with shRNA #1 survived, Figure 7G), and the phenotype had progressed with strong rightward skewing (white arrowheads), failed turning, and an open abdominal wall with an externalized liver (Figure 7H). Survival rates dropped between E9.5 and E13.5, with 0% survival at E18.5, suggesting that the absence of *Sptbn2* is embryonic lethal and crucial for neural tube development, embryonic turning, and abdominal wall closure.

Axial skewing and abdominal wall defects have previously been described for compound mutants with disruption in planar cell polarity components *Scrib*, *Celsr1*, or *Vangl2* (Murdoch et al., 2014), which govern the process of convergent extension and bending along the mediolateral axis of the neural tube (reviewed in Nikolopoulou et al., 2017). To test whether *Sptbn2* knockdown affects neurulation via related mechanisms, we further analyzed the *Foxa2* staining at E9.5 in *Sptbn2* shRNA virus-transduced embryos (Figure 7I). Sections from thoracic to sacral levels revealed a wider floorplate region (red arrows in Figure 7I, boxed regions) which was more pronounced in lumbar sections, similar to the floorplate widening seen in compound planar cell polarity (PCP) mutants (Murdoch et al., 2014). In Class 3 embryos, the sacral spinal cord was open. In sum, NEPTUNE achieved strong downregulation of *Sptbn2* mRNA and a dose-dependent phenotype severity correlating with the efficiency of SPTBN2 protein downregulation. *Sptbn2* silencing induced defects in neural tube, embryonic turning, and abdominal wall closure, which mimicked the phenotype of PCP mutants (Murdoch et al., 2014). Furthermore, the data show that *Sptbn2* loss of function is incompatible with life and suggest that previous models of *Sptbn2* mutations might reflect hypomorphic models.

## Discussion

Ultrasound-guided *in utero* nanoinjection has proved to be a powerful tool to unravel genetic networks in skin (Beronja et al., 2010, 2013; Schramek et al., 2014). Here, we show that the developing mouse nervous system can be targeted in a highly efficient and reproducible manner, achieving over 95% transduction efficiency throughout the brain and spinal cord (Figures 1, 2, and 3). Transduction is stable to adulthood (Figure 1). Using MiniPromoter sequences enabled expression in defined cell types without the need for transgenic *Cre* mouse lines (Figures 4, 5, S4, and S5). Proof-of-principle experiments demonstrated that NEPTUNE could recapitulate the published *Olig2*^−/−^ phenotype (Takebayashi et al., 2002) (Figure 6). In addition, we demonstrated that knockdown of *Sptbn2 in vivo* results in dose-dependent defects including embryonic lethality by E18.5, abdominal wall defects and a skewed body axis at E13.5, and embryonic turning defects with a widened floorplate at E9.5 (Figure 7). Our data thus reveal a function for this gene in neurulation (Figure 7), likely via PCP-related neurulation mechanisms, which warrants further investigation.

NEPTUNE allows high efficiency and widespread transduction of the nervous system. We identified volumes injected, viral titer, and freshness of the virus as crucial determinants of transduction and survival efficiency (Figures 1 and S1), and determined the optimal parameters to achieve >95% transduction in brain and spinal cord. To achieve conditional expression, we replaced the PGK promoter with MiniPromoters (Portales-Casamar et al., 2010) for *DCX*, *GFAP*, and *OLIG1*. Conditional gene expression is typically achieved by using Cre mice, but these mice are often generated by random *Cre* integration and less frequently by knocking *Cre* into the locus of a cell-type-specific gene. Importantly, random *Cre* integration is associated with DNA rearrangements around the integration site, inclusion of unreported genetic sequences, and multiple integrations (Cain-Hom et al., 2017). Furthermore, CRE expression itself can be toxic *in vivo* (Pomplun et al., 2007) and can induce DNA damage via endogenous cryptic *LoxP* sites (Loonstra et al., 2001), confounding interpretation of phenotypes obtained with Cre mice. Finally, CRE-mediated regulation of gene expression also entails a delay between conditional transcription of *Cre*, translation of CRE, and *LoxP* site editing, leading ultimately to expression of the conditional sequence of interest. This delay can be important when studying early developmental phenotypes. The MiniPromoters displayed robust expression, with no leakiness in SOX2^+^ precursor cells (Figures 4I–4K), but had variable efficacy in labeling neurons, astrocytes, and oligodendrocytes. Whereas the *DCX-H2B-GFP* construct was specifically active in neurons (Figure 4I, 4L–4N, S4A, and S5) and *GFAP-H2B-GFP* was expressed specifically in astrocytes (Figures 4J and S4B), the *OLIG1-H2B-GFP* could be observed in OLIG1-negative cells (Figure S4C). This might reflect transient *Olig1* expression in glial precursors contributing to astrocytes and oligodendrocytes, in which expression is downregulated during differentiation into astrocytes, whereas the GFP reporter is stabilized via fusion to H2B and might be expressed longer than OLIG1. Conditional expression with NEPTUNE with MiniPromoters is thus possible, but each promoter construct should be tested and validated. Future work to combine NEPTUNE with, for example, DIO systems (Double-floxed Inverted Open reading frame, also known as Flex switches [Schnütgen et al., 2003]) could further extend the toolbox for achieving conditional expression by using Cre mice of interest.

Previous work established that *in utero* transduction could target skin and has been used to elucidate mechanisms of development and disease in skin (Beronja et al., 2010, 2013; Schramek et al., 2014). These injections generally utilize injection of 1 μL at E9.5, a stage at which the amniotic cavity is larger and the embryo is easy to identify on ultrasound (Figure S1A). Gaiano et al. (1999) showed, over 20 years ago, that E8.5 amniotic cavity injection could target the nervous system, but transduction efficiency was uneven and E8.5 injection yielded 41% survival, of which 41% of embryos exhibited exencephaly, likely due to the high volumes injected. Here, we adapted the technique to injection at E7.5 to target the entire neural plate and NMPs contributing to the future nervous system. Survival of E7.5 virus-injected embryos, with optimal conditions, ranged from 40% to 90% (e.g. Figure 7G) and did not lead to brain malformations under these conditions.

Future modifications to NEPTUNE could increase its versatility. The rise of AAV evolution (Chan et al., 2017; Davidsson et al., 2019) and, thus, engineering viral tropism, might allow targeted transduction of specific cells during embryonic development. The insertion of a large promoter sequence reduced viral titer obtained from virus production by about 50%, as expected (Kumar et al., 2001) (viral titers described in STAR Methods); therefore, improved systems for virus production or packaging would also further strengthen the power and versatility of this technique.

The development of NEPTUNE can reduce the number of mice used in research. It has been estimated that of 3,872 targeted genes in the mouse, 45% are not essential for viability or fertility (Liao and Zhang, 2007). Conversely, 14% of mutated genes lead to embryonic lethality, usually at or before mid-gestation (Liao and Zhang, 2007; Wilson et al., 2005). Combining these two, the probability of knocking out a gene, and obtaining knockout mice to study that exhibit an interesting and important phenotype during embryogenesis, is around 40%. The development of NEPTUNE might allow screening for relevant neural phenotypes and circumvents the risk of severe embryonic lethality in heterozygous mice. Furthermore, NEPTUNE could be used to investigate genetic redundancy and dissect signaling networks. As an example, breeding triple heterozygous mice to obtain triple knockouts would generate 64 pups to obtain one wild type and one triple knockout. With NEPTUNE, it would thus be possible to directly generate the genotypes/knockdowns of interest, avoiding waste. Furthermore, validation of gene function across different strains of mice is facilitated, given that back-crossing would not be required, further improving and facilitating reproducibility.

The *Sptbn2* phenotype (Figure 7) is more severe than expected, based on our current knowledge of *SPTBN2* in the human ataxic syndromes SCA5 and SCAR14. However, given its role as a cytoskeletal component linking actin and the cell membrane (Liem, 2016), it is perhaps less surprising that disruption of *Sptbn2* would lead to neural tube defects and defects in embryonic turning. Importantly, in most patients, the described mutations are missense or in-frame deletions, suggesting that remnant protein might be sufficient to execute some SPTBN2 functions (Cho and Fogel, 2013; Elsayed et al., 2014; Ikeda et al., 2006; Lise et al., 2012; Nicita et al., 2019; Ranum et al., 1994; Wang et al., 2014; Yıldız Bölükbaşı et al., 2017). Likewise, the *Sptbn2* mutant mice still express slightly shorter forms of SPTBN2, suggesting some functional rescue (Perkins et al., 2010; Stankewich et al., 2010). Our data, using two different shRNAs, suggest that *Sptbn2* also has a crucial role in neural development and embryonic turning. With respect to the skewed body axis and abdominal wall closure defects, the *Sptbn2*-knockdown phenotype mimics PCP mutants with single or compound mutations in *Scrib*, *Celsr1*, or *Vangl2* (Murdoch et al., 2014), suggesting that Spectrin might play a key role in mediating PCP programs during neurulation and turning. However, PCP mutants generally have an extensively open neural tube, which was not present in any of the *Sptbn2*-knockdown embryos, highlighting a key difference.

In sum, NEPTUNE is a powerful technique to modulate gene expression during embryonic development. It can achieve widespread, stable, and conditional expression in the brain and spinal cord, and can be used to reveal roles for genes in crucial embryonic processes.

### Limitations of the study

Caveats for NEPTUNE with shRNA knockdown include the risk of variable knockdown, as seen in Figure 7, off-target effects due to lentivirus integration, and variable integration copy number. Variable knockdown can present advantages and disadvantages. At E9.5, 67 of 99 *Sptbn2* knockdown embryos survived (68%), but at E13.5 only 2 of 6 (33.3%) embryos survived. The open abdominal wall defect present at E13.5 (Figure 7H) is likely presented by the only surviving Sptbn2 embryos, and thus likely most mildly affected Class 1 embryos. This phenotype would have been missed if all embryos manifested strong knockdown and died prior to E13.5. Future work comparing shRNA knockdown and CRISPR editing of the corresponding gene should be undertaken to compare phenotypes upon knockdown and genetic perturbation. Genetic compensation in genetically perturbed organisms might mask critical functions (Rossi et al., 2015), and it will thus be of great interest to compare traditional knockouts, NEPTUNE-mediated CRISPR gene editing, and NEPTUNE-mediated shRNA knockdown.

Although it is possible to achieve widespread (up to 99%) and stable (tested up to 6 months) transduction of the developing mouse brain with NEPTUNE, we could not reach 100% of the cells in the future brain, and similarly current efficiency for spinal cord was a maximum 79%. Lentiviral packaging capacity also limits the production of high-titer lentivirus, as exemplified by a 50% reduction in titer when using MiniPromoters to drive GFP expression. Although NEPTUNE can achieve cell-type-specific effects without dedicated Cre mice, combination of NEPTUNE with Cre or Cas9 mice would further improve conditionality or capacity to knock genes out while allowing for smaller lentiviral loads.

## STAR★Methods

### Key resources table

**Table:** 

REAGENT or RESOURCE	SOURCE	IDENTIFIER
**Antibodies**		

Rabbit monoclonal anti-beta Actin	Cell Signaling	8457; RRID: AB_10950489
Mouse monoclonal anti Calbindin D-28k	Swant	300; RRID: AB_10000347
Rabbit polyclonal anti-Casp3	Cell Signaling	9961; RRID: AB_10697500
Guineapig polyclonal anti-Dcx	Millipore	Ab2253; RRID: AB_1586992
Chicken polyclonal anti-GFP	Abcam	Ab13970; RRID: AB_300798
Rabbit polyclonal anti-Gfap	Agilent	Z0334; RRID: AB_10013382
Mouse monoclonal anti-Gapdh	Sigma-Aldrich	G8795; RRID: AB_1078991
Mouse monoclonal anti-Hb9	DSHB	81.5C10; RRID: AB_2145209
Mouse monoclonal anti-Islet-1	DSHB	39.4D5; RRID: AB_2314683
Mouse monoclonal anti-NeuN	R&D Systems	MAB377; RRID: AB_2298767
Mouse monoclonal anti-Olig1	R&D Systems	MAB2417; RRID: AB_2157534
Goat polyclona anti-Olig2	R&D Systems	AF2418; RRID: AB_2157554
Rabbit monoclonal anti-Pdgfra	Cell Signaling	3174; RRID: AB_2162345
Goat polyclonal anti-Sox2	Santa Cruz	Sc-17320; RRID: AB_2286684
Mouse monoclonal anti-Sptbn2	Abcam	Ab238055
Alexa Fluor 488 AffiniPure Donkey Anti-Chicken IgY (IgG) (H+L), secondary antibody	Jackson Immuno Research	AB_2340375; RRID: AB_2340375
Donkey anti Goat IgG (H+L) Secondary Antibody, Alexa Fluor 546	Thermo Fisher Scientific	A11056; RRID: AB_142628
Donkey anti Goat IgG (H+L) Secondary Antibody, Alexa Fluor 647	Thermo Fisher Scientific	A21447; RRID: AB_141844
Goat anti-Guinea Pig IgG (H+L) Secondary Antibody, Alexa Fluor 546	Thermo Fisher Scientific	A11074; RRID: AB_1500609
Donkey anti Mouse IgG (H+L) Secondary Antibody, Alexa Fluor 546	Thermo Fisher Scientific	A10036; RRID: AB_2534012
Donkey anti Mouse IgG (H+L) Secondary Antibody, Alexa Fluor 647	Thermo Fisher Scientific	A31571; RRID: AB_162542
Donkey anti Rabbit IgG (H+L) Secondary Antibody, Alexa Fluor 546	Thermo Fisher Scientific	A10040; RRID: AB_2534016
Peroxidase AffiniPure Goat Anti-Mouse IgG (H+L)	Jackson Immuno Research	AB_10015289; RRID: AB_10015289
Peroxidase AffiniPure Goat Anti-Rabbit IgG (H+L)	Jackson Immuno Research	AB_2307391; RRID: AB_2307391

**Chemicals**, **peptides**, **and recombinant proteins**		

DMEM, high glucose, GlutaMAX™ Supplement, pyruvate	Gibco, Thermo Fisher	10569010
Fetal Bovine Serum, qualified, heat inactivated, Brazil	Gibco, Thermo Fisher	10500064
Penicillin-Streptomycin (10,000 U/mL)	Gibco, Thermo Fisher	15140122
Geneticin™ Selective Antibiotic (G418 Sulfate) (50 mg/mL)	Gibco, Thermo Fisher	10131027
MEM, GlutaMAX™ Supplement	Gibco, Thermo Fisher	41090036
Hexadimethrinbromid	Sigma/Merck	H9268
Isofluran	Pharmacy/Baxter	50085412586613
Hair removal crème	Pharmacy/Veet	05701092103888
Buprenorphine/Temgesic	Pharmacy	5054792001955
Occulentum Simplex Eye gel	Pharmacy/APL	07322833361640
DPBS, no calcium, no magnesium	Gibco, Thermo Fisher	14190169
37wt% formaldehyde (with 10-15% methanol)	Sigma/Merck	F1635
Sucrose	Sigma/Merck	S5016
OCT Cryomount	Histolab	45830
Tween20	Sigma/Merck	P9416
Donkey Serum	Sigma/Merck	D9663
Sheep serum	Sigma/Merck	S3772-10ML
Fluoroshield Mounting Medium	Sigma/Merck	F6182
Hydromount	Electron Microscopy Sciences	17966
DAPI	Sigma/Merck	D9542
Fast Sybrgreen Master Mix	Thermo Fisher	4385612
Trypsin 0.25% EDTA	Thermo Fisher	25200056
L-Cystein	Sigma, Merck	C-7880
Beta-mercaptoethanol	Sigma, Merck	M3148
Papain enzyme	Sigma, Merck	P4762
EDTA (0.5 M), pH 8.0, RNase-free	Invitrogen, Thermo Fisher	AM9260G
2M Calcium Chloride	Fisher Scientific	BP742
EcoRI-HF	NEB	R3101L
SmaI	NEB	R0141S
BamHI-HF	NEB	R3136L
NheI	NEB	R3131S
FseI	NEB	R0588S
XmnI	NEB	R0194S
SacII Enzyme	NEB	R0157
SphI-HF	NEB	R3182
NdeI	NEB	R0111S
SalI-HF	NEB	R3138S
CutSmart Buffer	NEB	B7204S
Calf intestinal alkaline phosphatase (CIP)	NEB	M0525S
T4 DNA Ligase	NEB	M0202S
UltraPure™ Agarose	Invitrogen, Thermo Fisher	16500100
One Shot™ Stbl3™ Chemically Competent E. coli	Invitrogen. Thermo Fisher	C737303
Terrific Broth	Sigma/Merck	T0918
Carbenicillin	Sigma/Merck	C3416
Ampicillin	Sigma/Merck	A5354
LB Broth with agar (Lennox)	Sigma/Merck	L2897
Nuclease free H_2_O	Sigma/Merck	W4502-1L
Lithium chloride for molecular biology, ≥99%	Sigma/Merck	L9650-100G
Methanol BioReagent, ≥99.93%	Sigma/Merck	494437-1L
Formaldehyde solution for molecular biology, 36.5-38% in H_2_O	Sigma/Merck	F8775-25ML
Glycine BioUltra, for molecular biology, ≥99.0% (NT)	Sigma/Merck	50046-50G
SSC Buffer 20× Concentrate	Sigma/Merck	S6639-1L
Ethanol 70%	VWR	83801.410
Ethanol 100%	VWR	20821.296
Deoxyribonucleic acid from herring sperm	Sigma/Merck	D7290-1ML
Magnesium chloride solution for molecular biology, 1.00M ± 0.01 M	Sigma/Merck	M1028
Sodium chloride for molecular biology, DNase, RNase, and protease, none detected, ≥99% (titration)	Sigma/Merck	S3014
Potassium chloride for molecular biology, ≥99.0%	Sigma/Merck	P9541
Hydrogen Peroxide Solution 30% (w/w)	Sigma/Merck	31642-500ML-M
Proteinase K	Sigma/Merck	1073930010
Glutaraldehyde solution 50 wt. % in H_2_O	Sigma/Merck	340855-25ML
Formamide, BioUltra, for molecular biology, ≥99.5% (T)	Sigma/Merck	47671-250ML-F
Heparin sodium salt from porcine intestinal mucosa	Sigma/Merck	H3393-10KU
Ribonucleic acid, transfer from baker's yeast (S. cerevisiae)	Sigma/Merck	R8759-100UN
Ribonuclease A from bovine pancreas for molecular biology	Sigma/Merck	R6513-10MG
Levamisole hydrochloride	Sigma/Merck	L9756-10G
Anti-Digoxigenin-AP, Fab fragments	Sigma/Merck	11093274910
NBT/BCIP Stock Solution	Sigma/Merck	11681451001
Sodium dodecyl sulfate solution BioUltra, for molecular biology, 20% in H_2_O	Sigma/Merck	05030-500ML-F

**Critical commercial assays**		

LookOut® Mycoplasma PCR Detection Kit	Sigma/Merck	MP0035
Quick Ligation Kit	NEB	M2200
QIAquick Gel Extraction Kit	Qiagen	28704
Phusion Green High-Fidelity DNA Polymerase PCR	Thermo Scientific	F534S
SP6/T7 Transcription Kit	Sigma/Merck	10999644001
DIG RNA Labeling Mix	Sigma/Merck	11277073910
PureLink™ Quick Plasmid Miniprep Kit	Invitrogen, Thermo Fisher	K210011
PureLink™ Expi Endotoxin-Free Maxi Plasmid Purification Kit	Invitrogen, Thermo Fisher	A31217
Maxima First strand cDNA kit	Thermo Scientific	K1641
PureLink™ RNA Mini Kit	Thermo Scientific	12183018A
PLUS Western Blot Stripping Buffer	Thermo Scientific	46430
SuperSignal™ West Femto Maximum Sensitivity Substrate	Thermo Scientific	34094

**Experimental models**: **cell lines**		

NE4C	ATCC®	CRL2925™
Lenti-X™ 293T	Clontech	632180

**Experimental models**: **organisms/strains**		

CD-1® IGS Mouse	Charles River, Germany	Crl:CD1(ICR)

**Primers**		

Primers used in this study are listed in Table S1	This paper	N/A

**Recombinant DNA**		

*Foxa2* ISH plasmid	Kind gift from Johan Ericson lab, Karolinska Institutet	
LV-RFP	Elaine Fuchs/Addgene	# 26001
LV-GFP (*hPGK-H2b-GFP*)	Elaine Fuchs/Addgene	# 25999
pEMS1172 (Ple151/*OLIG1*)	Elizabeth Simpson/Addgene	#29301
pEMS1199 (Ple53/*DCX*)	Elizabeth Simpson/Addgene	#29100
pEMS1375 (Ple88/*GFAP*)	Elizabeth Simpson/Addgene	#29176
psPAX2	Didier Trono/Addgene	#12260
pMD2.G	Didier Trono/Addgene	#12259
Scramble shRNA	David Sabatini/Addgene	#1864
*DCX-H2B-GFP*	This study	*DCX-H2B-GFP*
*GFAP-H2B-GFP*	This study	*GFAP-H2B-GFP*
*OLIG1-H2B-GFP*	This study	*OLIG1-H2B-GFP*
*Sptbn2* shRNA #1	Mission shRNA, Sigma	NM_021287.1-1398s21c1
*Sptbn2* shRNA # 2	Mission shRNA, Sigma	NM_021287.1-6582s21c1
*Sptbn2* shRNA #3	Mission shRNA, Sigma	NM_021287.1-1398s1c1
*shSptbn2* shRNA #4	Mission shRNA, Sigma	NM_021287.1-6582s1c1
*shSptbn2* shRNA #5	Mission shRNA, Sigma	NM_021287.1-5365s1c1
*shOlig2* shRNA #1	Mission shRNA, Sigma	NM_016967.2-624s1c1
*shOlig2* shRNA #2	Mission shRNA, Sigma	NM_016967.2-441s1c1
*shSptbn2-H2B-GFP #1* LV-GFP containing *Sptbn2* shRNA #1 NM_021287.1-1398s21c1, cloned as described	This study	*shSptbn2-H2B-GFP #1*
*shSptbn2-H2B-GFP #2* LV-GFP containing *Sptbn2* shRNA # 2 NM_021287.1-6582s21c1, cloned as described	This study	*shSptbn2-H2B-GFP #2*
*shOlig2-H2B-GFP #1* LV-GFP containing *shOlig2* shRNA #1 NM_016967.2-624s1c1, cloned as described	This study	*shOlig2-H2B-GFP #1*
*shOlig2-H2B-GFP #2* LV-GFP containing *shOlig2* shRNA #2 NM_016967.2-441s1c1 cloned as described	This study	*shOlig2-H2B-GFP #2*

**Software and algorithms**		

Primer3 web version 4.1.0	https://primer3.ut.ee/	N/A
GraphPad Prism 9	GraphPad Software	N/A
ZEN 3.2 ZEN lite	Zeiss	N/A
Image Lab 6.0.1	BioRad	N/A
Cell Profiler 3.1.9	CellProfiler	N/A
“Counting and Scoring” Cell Profiler Pipeline	CellProfiler	https://cellprofiler.org/examples

**Other**		

Cotton Swab	Medicarier	60406
Ultrasound gel	Medexa	N/A
Suture for mice: J384H, Vicryl, 6-0, C-3 needle, 45cm purple filament	Agnthos	J384H
Iris Scissors, Super Cut, straight, 9 cm	Agnthos	307-336-090
Dressing forceps delicate straight 13 cm	Agnthos	08-032-130
Vevo2100	Visual Sonics	VS-20047
70MHz MS Series transducer	Visual Sonics	MS700
Vevo Imaging Station 2	Visual Sonics	VS-11983
Mouse Handling Table	Visual Sonics	50249
Vevo Compact Dual (Med. Air & O2) Anesthesia System	Visual Sonics	VS-12055
Nanoject II Auto Injector Kit	Drummond	3-000-205A
Steri 250, hot bead sterilizer	Angthos	31100
EG-400 Narishige Micropipette Grinder	Narishige	N/A
Petri dish with central opening (low wall)	Visual Sonics	SA-11620
Silicone membrane	Visual Sonics	SA-11054
EZ clips 9 mm	Angthos	59021
Superfrost Plus Slides	VWR	631-9483
Hydrophobic Barrier PAP Pen	Vector Labs	H4000
Corning Square bioassay dishes	Sigma/Merck	CLS431110
Sealtape for 96-well plate	Thermo Scientific	232698
PVDF membrane 45μm	Thermo Scientific	88518

**Other**		

Deposited data for this manuscript can be found at the specified link	This study	https://doi.org/10.17632/rr6c34zgsw.1

### Resource availability

#### Lead contact

Further information and requests for resources and reagents should be directed to and will be fulfilled by the lead contact, Dr. Emma R. Andersson (emma.andersson@ki.se).

#### Materials availability

All newly generated items used in this study are available upon request and following standard Material Transfer Agreement (MTA), due to institutional recommendations.

#### Data and code availability

This study did not generate computer algorithms or codes.

All data available in the main text, supplementary materials, and raw data are deposited in Mendeley Data at https://doi.org/10.17632/rr6c34zgsw.1.

### Experimental models and subject details

#### Animals

CD1 wild type mice were obtained from Charles River Laboratories (Germany). Animals were housed according to European regulations, with a standard day and night cycle with food and water ad libitum. From the age of 8 weeks, females were checked for estrus and plugged overnight. Gestation was defined as embryonic day (E) 0.5 at noon of the same day of vaginal plug. Ethical approval for all experiments described here was granted by the Swedish Board of Agriculture (Jordbruksverket) with permit numbers N59/14, 8188-2017 and 2987-2020.

#### Cell lines and culture conditions

Lenti-XTM 293T cells (Clontech) were grown at 37°C/ 5% CO_2_ in DMEM complete medium (DMEM, high glucose, GlutaMAX, pyruvate supplemented with 10% fetal bovine serum, 1% penicillin/streptomycin and 1% geneticin). For virus production, low passage cells (p4-p6) were thawed and seeded into a 75cm^2^ cell culture flask one week prior to virus production. Upon transfection with plasmids for virus production, DMEM complete medium was replaced with DMEM virus medium for the rest of the process (DMEM, high glucose, GlutaMAX, pyruvate Supplement with 10% fetal bovine serum, 1% penicillin/streptomycin).

NE4C cells (ATCC) were grown until passage 15 at 37°C/ 5% CO_2_ in MEM complete medium (MEM, GlutaMAX supplemented with 10% fetal bovine serum and 1% penicillin/streptomycin). Upon transduction with lentivirus for titration experiments, MEM complete medium was replaced by MEM infect medium (MEM, GlutaMAX supplemented with 10% fetal bovine serum and 0.1 mg/mL Polybrene).

Both Lenti-XTM and NE4C cell lines were free from mycoplasma contamination and underwent regular screening for mycoplasma.

### Method details

#### Cloning of shRNA constructs

5 μg of plasmid, containing shRNA (TRC, Sigma Aldrich) or scrambled control shRNA (gift from David Sabatini) or *hPGK-H2B-GFP* reporter (gift from Elaine Fuchs, also known as *LV-H2B-GFP*) were cut with SacII and SphI for 15 min to 1 h at 37°C in CutSmart buffer. (Vector backbone for all plasmids is pLKO.1). SphI cuts within the 5’ LTR and SacII cuts just upstream of the *hPGK* promoter. Digests were run on a 1% Agarose gel at 100 V for 1 h. For shRNA or scrambled control shRNA digests (insert), the band at 2243 bp (containing the U6 promoter, shRNA insert and 5`LTR), and for *GFP* reporter digests (vector) the band at 5263 bp (containing the *hPGK* promoter, the *H2B-GFP* reporter and the 3`LTR) were excised and DNA was extracted using the Qiagen QuickGel extraction kit. Ligations were set up with a molar ratio 3:1 of insert:vector. Using NEB Quick ligation kit, ligation mixture was incubated at room temperature for 5 min. The reaction was put on ice and One Shot Stbl3 chemically competent cells were transformed following the manufacturers' protocol. 100 μL transformation mix was spread on an Agar plate containing 100 μg/mL Ampicillin, and the plate was incubated overnight at 37°C. The next day, 3–5 colonies were picked for screening and incubated on a shaker in 2 mL Terrific Broth containing 100 μg/mL Ampicillin for 8 h. Plasmid DNA was extracted using PureLink Quick Plasmid Miniprep Kit. A diagnostic digest was performed using NdeI and SacII. Vectors were validated by sequencing using the Eurofins barcode sequencing service.

#### Cloning MiniPromoters

The *LV-MiniP-H2B-GFP* vectors (*DCX-H2B-GFP*, *GFAP-H2B-GFP* and *OLIG1-H2B-GFP*) were prepared by inserting the respective MiniP sequences (Portales-Casamar et al., 2010) into the *LV-H2B-GFP* vector (Beronja et al., 2010) using PCR-introduced SalI and FseI restriction sites. Specifically, the *hPGK* promoter was removed from the *LV-H2B-GFP* vector by PasI digest, the vector was dephosphorylated by CIP, and gel-purified using Qiagen QuickGel extraction kit. The promoter-less linear backbone plasmid, and MiniP containing pEMS1172, pEMS1375, and pEMS1199 plasmids (Portales-Casamar et al., 2010), were used as templates for SalI, FseI restriction site introduction by Phusion Green High-Fidelity DNA Polymerase PCR, using manufacturers protocol (30 cycles, 2-step protocol, no GC additive, 5% DMSO) and JM120F/R, JM121F/122R primers. Appropriate PCR products from pEMS1172 (3165 bp), pEMS1375 (2340 bp), pEMS1199 (3643 bp), and *LV-H2B-GFP* (6998 bp) were excised from the gel and gel-purified. The purified PCR fragments were digested with SalI/FseI restriction enzymes and the FseI containing *LV-H2B-GFP* backbone PCR fragment was dephosphorylated. Cleaved DNA fragments were immediately column-purified, ligated with T4 DNA ligase, and transformed into One Shot Stbl3 chemically competent E. coli. Resulting clones were screened by PCR (*OLIG1-H2B-GFP* - JM117F/R - 268bp, *GFAP-H2B-GFP* - JM118F/R - 208bp, *DCX-H2B-GFP* - JM119F/R - 236bp), restriction digest (*OLIG1-H2B-GFP* - EcoRI - products 7329 bp + 2755 bp, *GFAP-H2B-GFP* - products SmaI + BamHI - 8709 bp+492 bp, *DCX-H2B-GFP* - EcoRI + NheI - products 7973bp + 1667bp + 875bp), and verified by Sanger sequencing.

#### Endotoxin-free plasmid prep

Bacteria containing the plasmids of interest were grown in 100 mL (for high copy plasmids) and 200 mL (for low copy plasmids) of Terrific Broth containing 100 μg/mL Carbenicillin, on a horizontal shaker for 20–22 h. Bacteria were centrifuged to pellet at 4000 g for 20 min at 4°C. Medium was discarded and bacterial DNA was extracted using PureLink™ Endotoxin-Free Maxi Plasmid Purification Kit, following the manufacturer’s instructions. Concentration was measured with a nanodrop.

#### Transfection

5∗10^4^ NE4C cells/well were seeded on 12-well plate 16–20 hours prior to transfection in MEM complete medium. Lipofectamine2000 and 1μg plasmid DNA were diluted in Opti-MEM to a final DNA:Lipofectamine ratio of 1:3 and incubated at room temperature for 5 min. Transfection Mix was added and incubated for 8 hours, before being replaced by fresh MEM complete medium. 24 or 48 hours aftertransfection, cells were collected for RNA or protein analysis (described further down under “RNA extraction and qPCR” or “Western Blot”).

#### Virus production

One 75 cm^2^ flask of LentiX-293T cells, at 80% confluency, was split into four 225 cm^2^ flasks. 24 hours prior to transfection, when cells were at 80%–90% confluency, one 225 cm^2^ flask was seeded 1:1 on one 500 cm^2^ plate (2 plates per virus were used, typically two viruses were produced at a time). On Day 0, cells were transfected using the calcium phosphate transfection method: 275 μg of vector plasmid, 275 μg of psPAX2 (packaging plasmid) and 180 μg of pMD2.G (VSV-G plasmid) were mixed in a 50 mL conical tube (pMD2.G and psPAX2 were a gift from Didier Trono). 2.28 mL of 2 M CaCl2 in MQ water were added to a final volume of 9.5 mL. Next, 9.5 mL 2xHBS (50 mM HEPES, 1.5 mM Na2HPO4, 280 mM NaCl, pH 7.07) was added and the tube was inverted 4 times. After incubation at room temperature for 60 seconds, the mixture was added to 165mL of pre-warmed DMEM virus medium (see above). 14–16 h after transfection, medium was replaced with fresh DMEM virus medium. Viral supernatant was collected at 46 h after transfection, and at 65 h. Upon collection, supernatant was filtered through a 0.45 μM Millipore low-protein binding filter units and kept at 4°C. Viral supernatant was concentrated by first using low-speed centrifugation through 100 kDa MW cutoff Millipore Centricon 70 Plus cartridges to condense the volume to < 4 mL, followed by ultracentrifugation through a 20% sucrose cushion at 45,000 rpm (MLS 50 Rotor) for 2 h, to pellet the lentiviral particles. That pellet was resuspended in 25–30 μL viral resuspension buffer (20mM Tris pH 8.0, 250 mM NaCl, 10 mM MgCl2 and 5% sorbitol) and 5 μL aliquots were stored at -80°C.

#### Virus titration

Viral titers for constructs containing the *hPGK-H2B-GFP* reporter were determined via spinfection of NE4C cells. 24 h prior to transduction, cells were seeded in MEM complete medium in a 6-well plate at a density of 5∗10^8^ cells/well. For calculation of the viral titer, the total number of cells per well, available for spinfection, must be determined. Therefore, on Day 0, one well was trypsinized and the cells were resuspended in 1.5 mL MEM complete medium. Cell number per mL volume was assessed using a Countess 3 Automated Cell Counter and adjusted for the total volume of 1.5 mL to obtain the total number of cells in one well. Medium was replaced with MEM infect medium in the remaining five wells. 5, 50, and 500 μL of virus diluted 1:2,000 and 1 μL of concentrated virus were added to four individual wells, one well was kept as non-transduced control. Plates were centrifuged for 30 min at 1100 g and 37°C. MEM infect medium was replaced with MEM complete medium and cells were incubated for 48 h at 37°C. The percentage of GFP+ cells was recorded using a Canto LSR II flow cytometer. In brief, cells were washed with PBS and trypsinized. Cell suspensions were collected in 1.5 mL tubes and spun for 5 min, 200 g. The cell pellet was washed and resuspended in PBS twice, before fixation in 4% formalin for 15min at room temperature. After two additional washing steps, the cell suspension was filtered through a 25μm cell strainer and kept on ice until analysis. This titration yields ifu/mL. For MiniPromoter constructs, NucleoSpin RNA virus kit and LentiX qRT-PCR Titration Kit were used. The manufacturer’s instructions were followed. qRT-PCR Titration yields viral particles/mL. Particles/mL was converted to ifu/mL based on comparisons of titers obtained with the two methods for the same viral preps, yielding a correction factor of 159 (159-fold higher particle titer compared to actual ifu/mL titer).

**Table:** 

Viral Titers	
*shSptbn2-GFP #1*	2.10∗10^10^ ifu/ml
*shSptbn2-GFP #2*	2.02∗10^10^ ifu/ml
*shOlig2-GFP #1*	2.09∗10^10^ ifu/ml
*shOlig2-GFP #2*	1.99∗10^10^ ifu/ml
*Scr-GFP Ctrl*	2.11∗10^10^ ifu/ml
*DCX-H2B-GFP*	9.28∗10^9^ ifu/ml
*GFAP-H2B-GFP*	9.51∗10^9^ ifu/ml
*OLIG1-H2B-GFP*	9.83∗10^9^ ifu/ml

#### Pregnancy verification by ultrasound and embryo staging

Pregnancy in plug-positive females was confirmed via ultrasound (US) the day before injection (verified at E6.5 for injections at E7.5, or at E7.5 for injections at E8.5). The pregnant female was placed in an induction box and anesthetized with an initial dose of 3%-4% Isoflurane. Once anesthetized, the female was moved to a pre-warmed (37°C) surgical table. To maintain anaesthesia, the snout was placed into a nose cone and the Isoflurane dose was lowered to 1.5%-2%. The female was placed on its back and all four paws were gently fixed to the table with surgical tape. The fur on the lower abdomen was removed by applying commercial Veet Hair Removal cream with a cotton swab. The abdomen was wiped clean with water to remove fur, and dried, before US-gel was applied. The US-probe was lowered into the gel and remained static, while the position of the female and table could be adjusted up/down, left/right using two wheels steering the surgical table. Once pregnancy was verified or refuted, US-gel was wiped off the abdomen, and the abdomen was cleaned with water. Confirmed pregnant females were placed into a separate, new cage and awakening was monitored over the first few minutes with a final check 15 minutes after removal from anesthesia.

Embryo number and amniotic cavity size for E7.5 embryos was assessed on the day of injection. The mouse was anesthetized following the same procedure as described above. Using ultrasound, the left and right uterine horns were completely scanned. The individual amniotic cavities were assessed in higher magnification images and recorded. Individual cavity sizes were not measured, and assessment was rapid and based on visual appearance only. The female that had been assessed was labelled with a pen mark on its tail and was put back into its cage. Once all the pregnant females had been staged, the surgical field was prepped, and injections started shortly after, or were postponed until a later time point, depending on embryo stage. However, if amniotic cavity size or embryonic stage were not sufficiently developed by 8pm (which should be slightly more than E7.75), injections were postponed to 5am the following day (which should be E8.25). In general, both pregnancy check and ultrasound staging were performed in under 10 min (from induction to awakening).

#### Needle loading and petri dish preparation

Glass capillaries were pulled and ground to a bevelled tip in-house, following the protocol of (Beronja et al., 2013). Needles were back-filled with mineral oil using a 1 mL syringe attached to a 25G needle. This also flushes the ground needle tip from any residual debris caused by the grinding. Prior to injection, needles were attached to the nanoinjector (Harvard Apparatus). The metal plunger was pushed out entirely, followed by loading a small amount of air into the needle, creating a small barrier in the form of a bubble between the mineral oil and the virus which was loaded thereafter. Virus (stored at -80°C) was brought to the injection room and kept on dry ice until loading. One vial was placed on wet ice, allowing the aliquot to thaw. The tube was centrifuged briefly in a table-top centrifuge and the entire volume (5μL) was transferred onto a piece of parafilm. The needle was lowered into the virus drop and was filled. Once the entire drop was loaded into the needle, an additional air bubble was created at the tip of the needle to prevent the fine tip from clogging. The nanoinjector with attached and loaded needle was always turned towards the back of the ventilated hood, away from the experimenter, when any other steps than injections themselves were performed.

Petri dishes with a 2x2 cm round hole in the bottom were purchased together with pre-cut pieces of silicon membrane (both Visual Sonics). The elastic membrane piece was glued to the exterior of the hole and a ca.1cm long incision was made in the membrane. During surgery, the exposed uterus is pulled through this elastic opening, so the size of the incision should be adjusted depending on the embryonic stage to be injected.

#### Surgery and ultrasound-guided nanoinjections

Anaesthesia induction and maintenance as well as animal placement as described above. Both eyes were covered with eye gel to prevent drying and 0.1 mg/kg Buprenorphine pain killer was injected subcutaneously. With a pair of surgical scissors, a 1–2 cm vertical midline incision was made in the lower abdomen. Two cuts were made, one to open the skin and one to open the muscle layer underneath. With two pairs of surgical forceps, both uterine horns were carefully exposed, and the total number of embryos was recorded. Embryos were counted from the ovary to the cervix. For the injections, only the 3–4 embryos nearest the ovary of the left or right uterine horn were left exposed, while the remaining embryos were carefully pushed back into the abdominal cavity with a sterile cotton swab. The exposed embryos were pulled through the elastic membrane of the modified petri dish. Commercial play dough was used to fashion four feet to secure the petri dish on the surgical table above the female. The dish was then filled with sterile PBS until the embryos were immersed. To prevent leakage, the elastic bottom was pushed down with a cotton tip, so that it adhered to the wet, surrounding skin. An additional piece of play dough was placed on the needle-averted side of the embryos, in order to immobilize them and to prevent any unwanted movement during injections. The US-probe and needle were lowered into the PBS and aligned with the first embryo, by moving the table via the wheels. An image of the amniotic cavity was taken, and the diameter was measured, which allowed for calculation of the maximum volume that could be injected without causing resorption. The needle should be inserted through the uterine wall into the amniotic cavity in one single movement. After injection of the desired volume, the needle was kept inside the amniotic cavity for an additional five seconds to allow the entire volume to exit the needle and avoid spilling of virus out of the amniotic cavity upon withdrawal of the needle. Then the needle was removed, again in one single movement. Once all embryos were injected, the US-probe and needle were lifted out of the PBS and the stabilizing playdough was removed. Once the last embryo was injected and put back into the abdomen, the muscular layer was sutured using 6-0 prolene and the skin was closed with EZ-clips. The female was placed into a cage on a 37°C heating mat and recovery was carefully monitored, with an additional check 15–30 min after surgery. All work was carried out in a laminar airflow hood. In between mice, all surgical instruments (forceps, scissors etc.) were sterilized in a 200°C glass bead sterilizer. Disposable products (cotton tips, tissues) were replaced with fresh ones.

#### Tissue collection and fixation

Pregnant female mice were sacrificed in a CO_2_ chamber. Whole uterine horns were exposed, and embryos were dissected out under a microscope in sterile, room-temperature PBS. Tissues were fixed in 4% formalin in PBS and dehydrated with a 30% sucrose buffer. Samples were embedded in OCT freezing medium on dry ice and stored at −80°C. 12 μm cryosections were prepared at −20°C on SuperFrost+ glass slides. Sections were kept at −20°C for short term storage, or −80°C for long-term storage.

**Table:** 

Embryonic/Adult stage	Time in Fixative
E9.5 whole embryo	20 min, room temperature
E10.5 whole embryo	1.5 h, 4°C
E13.5-E14.5 head and body separated	4–5 h, 4°C
E18.5 brain	overnight, 4°C
6 months brain	overnight, 4°C

#### Immunohistochemistry

Glass slides were thawed at room temperature and the edges of the sections were outlined with a PAP Pen to create a hydrophobic barrier. Sections were re-hydrated in PBS for 5–10 min and incubated with blocking buffer (5% Donkey Serum in 0.3% PBS-Tween) for 30 min to 1 h at room temperature. Primary Antibody was diluted in blocking buffer and incubated on slides overnight at 4°C. The following day, the slides were washed 3x5 min in PBS. Secondary Antibody, diluted in blocking buffer, was added and incubated for 1 h at room temperature. Washing was repeated (3x5 mins) and slides were mounted with Fluoroshield mounting medium and stored at 4°C. Images were acquired with an LSM880 confocal microscope. Primary Antibody dilutions used: Calb1 (1:2,000); Cleaved Caspase-3 (1:400); GFP (1:1,000); Gfap (1:1,000); Hb9 (1:20); Islet-1 (1:50); NeuN (1:200); Olig1 (1:100); Olig2 (1:200); Pdgfra (1:1,000); Sox2 (1:200); Sptbn2 (1:200). Secondary Antibodies were used 1:500.

#### RNA In situ hybridization (ISH)

Probe synthesis: The *FoxA2* containing plasmid, was linearized using XmnI restriction enzyme (10 μg of *FoxA2* cDNA, 2 μl of XmnI, 5 μl Cut Smart Buffer, 33 μl nuclease free H_2_O); 37°C/1h), gel purified (Qiagen, elution in 50μl of nuclease free H_2_O) and used for the synthesis of the anti-sense probe using the DIG RNA labeling mix according to the manufacturer’s instructions (1 μg of linear *FoxA2* vector (13ul), 2 μl DIG mix, 2 μl of the Transcription buffer, 2 μl of T7 RNA polymerase, 1μl of nuclease-free H_2_O, 37°C/2h). The produced probe was precipitated using Ethanol by adding 2 μl of EDTA (0,2M), 2,5 LiCl (4M) and 75ul of 70% (v/v) EtOH, this solution was kept in -80°C/30 min, spun down at 13,000g/4°C/15min in a table top centrifuge. The resulting pellet was carefully washed with 100 uL 70% (v/v) EtOH, and centrifuged again at 13,000g/4°C/15min. The EtOH was carefully decanted, and the pellet was air dried at RT for 30 min, and dissolved with 50 μl of nuclease-free H_2_O. Probe yield was assessed by running 5 μl on a 1,5% agarose gel at 140V for 30 min. An additional 50 μl of nuclease-free H_2_O was added to the remaining 45 μl of the probe and probe was aliquoted into 9 tubes. 2.5 aliquots (= 22.5 μl of probe ) were used for each staining.

Embryo collection: Pregnant female mice carrying embryos injected 48hours before (E7.5) with the desired combination of viruses were sacrificed in a CO_2_ chamber. Whole uterine horns were exposed, and embryos were dissected out under a binocular microscope in sterile, ice-cold DPBS, all extra-embryonic tissue was removed.

RNA ISH: Unless stated otherwise, all steps are performed in 2 ml Eppendorf® LoBind microcentrifuge tubes, and the washes were performed with filtered 1ml blue tips.

Day 1: All solutions were RNAase-free. The freshly dissected embryos were moved to 2ml tubes (up to 7–8 per tube), and rinsed once with sterile, ice-cold DPBS, followed by 4% PFA (in sterile DPBS) fixation for at least 4h, optionally O/N at 4°C. Embryos were then dehydrated successively in 25%, 50%, 75%, 100% MeOH (in PBT - sterile DPBS + 0,1% Tween-20), 10 min/RT each for storage. Embryos can be thus stored at −20°C for several months.

When initiating ISH, the 100% Methanol was exchanged for Bleaching solution for 10min (MeOH+ H_2_O_2_ in a 4:1 ratio), followed by re-hydration with 50%, and 25% MeOH in PBT, 10 min/RT each. The last 25% MeOH in PBT wash is followed by 3x/5min/RT washes in PBT, followed by 10–12 min incubation in PBT + Proteinase K (10mg/ml) (caution: embryos are very fragile at this stage). The reaction was terminated using 2x 5 min/RT wash with glycine in PBT (30mM final concentration) followed by 2x 5min/RT wash with PBT. The embryos were then re-fixed with 0,2% glutaraldehyde + 4% PFA in PBT for 20 min/RT on a rocking platform and washed twice for 5min/RT washes in PBT.

PBT was then exchanged with 1,6 ml of pre-warmed (70°C) Prehybridization solution and incubated for 60-90min/70°C in a heating block.

The *FoxA2* anti-sense probes were denatured in PCR tubes in 100 μL of the Hybridization solution/ 80°C/10min. After the incubation, tubes with FoxA2 probes were immediately placed on ice and spun down if necessary. After the incubation of embryos in Prehybridization solution, 0,8 mL removed, and replaced with 0,7mL of pre-warmed Hybridization solution and 100ul of the denatured probe was added, without letting the solution cool down (in a heating block) and incubated O/N with gentle agitation at 70°C.

Day 2: Embryos were washed 2x 30min/70°C with 1,5 mL of SI, 1x 10min/70°C with SI+SII (1:1 ratio), 3x 5min/RT with SII, followed by 2x 30 min/37°C with SII + RNaseI (final concentration 10mg/mL), RNaseI-containing SII was removed and 2x 30min/65°C washes were perfomed with SIII, followed by 3x 10min/RT washes with TBST+ Levamisole (final concentration 2mM). Non-specific binding was blocked by 2h/RT incubation in heat-inactivated 10% sheep serum (in TBST), after which the tubes were left O/N/ 4°C with 1,5mL of anti-DIG solution (anti-DIG-alkaline phosphatase 1:2000 in 10% sheep serum (in TBST)) on a rocking platform.

Day 3: Embryos were washed 6x 1h/RT with TBST+2mM Levamisole on a rocking platform, followed by a longer O/N/ 4°C wash in the same solution.

Day 4: The buffer was removed and 2x 20min/RT washes were done with NTMT buffer, and the embryos were stained in the dark at RT (1-3h) with NBT/BCIP (1x from 50x concentrate) mixture in NTMT. When adequate signal was observed, the reaction was stopped with 3x 5min/RT washes with PBT + 1mM EDTA. Embryos were post-fixed with 4% PFA in PBS and kept in the fridge until imaged (up to several weeks).

**Table:** 

Prehybridization solution	Final concentration	Hybridization solution	Final concentration
Formamide	50%	Formamide	50%
20x SSC	5x	20x SSC	5x
Nuclease free H_2_O	-	Nuclease free H_2_O	-
EDTA 0,5M	5 mM	EDTA 0,5M	5 mM
10% Tween20	0,1%	10% Tween20	0,1%
Heparin 100mg/mL	50 μg/mL	Heparin 100 mg/mL	50 μg/mL
-	-	tRNA 10 mg/mL	50 μg/mL
-	-	herring sperm DNA 10mg/mL	50 μg/mL
**Solution SI**	Final concentration	**Solution SII**	Final concentration
Formamide	50%	5M NaCl	1M
20x SSC	5x	1M Tris-Cl, pH 7,5	10 mM
10% SDS	1%	10% Tween20	0.1%
Nuclease free H_2_O	top up	Nuclease free H_2_O	top up
**Solution SIII**	Final concentration	**TBST**	Final concentration
Formamide	50%	5M NaCl	130 mM
20x SSC	2x	1M Tris-Cl, pH 7,5	25 mM
Nuclease free H_2_O	top up	1M KCl	2,7 mM
**NTMT**	Final concentration	10% Tween20	0.1%
5M NaCl	100 mM	Nuclease free H_2_O	top up
1M Tris-Cl, pH **9**,**5**	100mM	-	-
1M MgCl_2_	50 mM	-	-
10% Tween20	0.1%	-	-
Nuclease free H_2_O	top up	-	-

Imaging of the embryos was performed on agarose-filled plates in DPBS using a stereomicroscope (Zeiss). After imaging of whole E9.5 embryos, embryos were cut in four pieces to facilitate transverse sectioning of spinal cord in embryos with different shape. For details on embryo partitioning used for embedding, please see Methods S1: Embryo partitioning for sectioning, related to STAR Methods. Pieces were embedded in OCT. Serial sections of 12 μm were collected on SuperFrost slides. The sections were rehydrated in PBS for 10–15 min, then mounted with Hydromount mounting media. Images were taken with an Axio Imager2 microscope, and 503 color camera.

#### Tissue dissociation

Papain enzyme was activated for 30 min at 37°C in 1x HBSS, supplemented with 1.1 mM EDTA, 0.067 mM beta-ME and 5.5mM L-Cystein. For E13.5 brain and E18.5 spinal cord, 5 units (U) of papain were used. 10 U were used for p1 brain, E13.5 whole embryos or p9 cerebellum. 200 U/mL DNaseI and 1 mM MgCl were added to the activated enzyme, and samples were incubated for 30 min at 37°C. Tissue was triturated two times in dissociation buffer (1xHBSS, 200 U/mL DnaseI, 1 mM MgCl) and centrifuged for 5 min, at 300g. The cell pellet was resuspended in ice-cold PBS. Cells were either analysed with a Canto LSR II flow cytometer, or GFP+ and GFP− cells were sorted using a FACSAria III. Cells were sorted into empty 15 mL falcon tubes at 4°C.

#### RNA extraction and qPCR

Sorted cells were centrifuged at 2,000 g for 5 min at 4°C to pellet and resuspended in Lysis Buffer containing β-Mercaptoethanol. NE4C cells were washed with ice cold PBS and lysed on ice with complete lysis buffer. Lysate was scraped with a pipette tip and collected in a chilled 1.5 mL tube. RNA was extracted on the same day using PureLink RNA Mini Kit, and cDNA was produced using RT-PCR kit (following manufacturer’s instructions). A cDNA standard of 5 concentrations with a dilution factor of 1:3 was prepared for each experiment. For qPCR, cDNA samples and standards were mixed with Fast SYBR Green Master Mix and the qPCR primers of interest and run for 40 cycles. Values were normalized to the housekeeping gene transcript *beta actin*, by dividing the relative amount of the target by the relative amount of *beta actin* per sample. In experiments involving GFP sorted and unsorted conditions, the values were additionally normalized to the average of the GFP− condition. Primer sequences were determined using Primer3 (Table S1) and checked for amplification across exon borders. In silico PCR confirmed specific target amplification.

#### Cell Profiler

Separate images of DAPI and GFP (488) from specific brain regions were exported to jpeg file format and loaded into Cell Profiler (see Key resources table for details). A ready-made pipeline from Cell Profiler was used (link in Key resources table), in which the cell nuclei were identified as primary objects in both images separately. The diameter range for the size of nuclei was set by measuring the size of several nuclei in one picture (output in pixel) and for calculating the threshold, Otsu and a two-class thresholding method was used. Finally, the number of nuclei in the GFP picture was divided by the number of nuclei in the DAPI picture to obtain the percentage of GFP+ nuclei per section.

#### Western blotting

E9.5 whole embryos or p10 cerebella were snap frozen and crushed with a mortar and pestle on dry ice. P9 cerebella were dissociated as described under “Tissue Dissociation”. The cell pellet or tissue powder was reconstituted in RIPA lysis buffer with Complete Protease inhibitor cocktail. NE4C cells were washed with ice-cold PBS and complete lysis buffer was added directly to the wells. Protein concentration was determined by Bradford assay. 25μg of sample was mixed with Laemli buffer and boiled at 95°C for 8 min. Samples were separated on a 4-20% MiniProtean precast gel and wet-transferred overnight at 30V to an Amersham Hybond 0.45 PVDF blotting membrane. Membranes were blocked in 5% non-fat milk in TBST (Tris-buffered saline + 0.1% Tween20) for at least 1h at room temperature and incubated in primary antibody overnight at 4°C. The following day, membranes were washed 3x15min in TBST at room temperature. Secondary antibody was added for 1 hour at room temperature, followed by 3x15 min washing. Membranes were developed with SuperSignal West Femto Substrate. The relative protein concentration was calculated in Image Lab using the individual band volumes. Membranes were rinsed in H_2_O to remove chemiluminescent substrate. For stripping and re-probing, the membrane was incubated in Restore Western Blot Stripping Buffer for 15 minutes at room temperature, followed by 3x15 min washing in TBST. Antibody incubation (primary and secondary) as well as blot development were performed as described above. Primary antibodies used were: Beta-actin (1:1,000); Gapdh (1:1,000) and Sptbn2 (1:200). Secondary antibodies were used 1:5,000.

### Quantification and statistical analysis

Quantification of GFP^+^ nuclei with Cell Profiler was performed on 20x tile images of brain or spinal cord sections. One section every 134μm was used for acquisition and quantification, resulting in 8-10 sections in total per embryo (covering fore- to hindbrain region or thoracic to sacral region in spinal cord). Figures 2G and S2C show the mean number of GFP^+^ nuclei in 2-4 sections per brain region from three different embryos, while in Figure S1E each data point represents the total mean of GFP^+^ nuclei per brain per embryo. In Figure S2 and 3A, every data point represents the number of GFP^+^ nuclei on one section.

Data were analysed using GraphPad Prism 9 and are presented as mean ± SD. When more than two groups were compared, a one-way ANOVA test was performed with the following parameters: No matching or pairing of replicates across groups, assuming normal distribution (Gaussian distribution) and assuming that groups do not have equal variances (Brown-Forsythe and Welch ANOVA test). In addition, multiple comparisons were done to assess differences between groups. To compare two groups only, a two-tailed Student’s t test was performed. Statistical method used is indicated in the individual figure legends. Statistical significance was defined as ∗p < 0.05, not significant was indicated as ns.

## Supplemental information

Document S1. Figures S1–S6 and Method S1

Table S1. Primers used in Mangold et al

Document S2. Article plus supplemental information
