# Supplementary material for: Highly efficient manipulation of nervous system gene expression with NEPTUNE
Source: Cell Rep Methods. 2021 Jul 6;1(4):100043. doi: 10.1016/j.crmeth.2021.100043 (PMC8457050; doi:10.1016/j.crmeth.2021.100043)
Supplement: Document S1. Figures S1–S6 and Method S1 [file mmc1.pdf]

**Cell Reports Methods, Volume *1***

## Supplemental information

# Highly efficient manipulation of nervous system gene expression with NEPTUNE

**Katrin Mangold, Jan Mašek, Jingyan He, Urban Lendahl, Elaine Fuchs, and Emma R. Andersson**

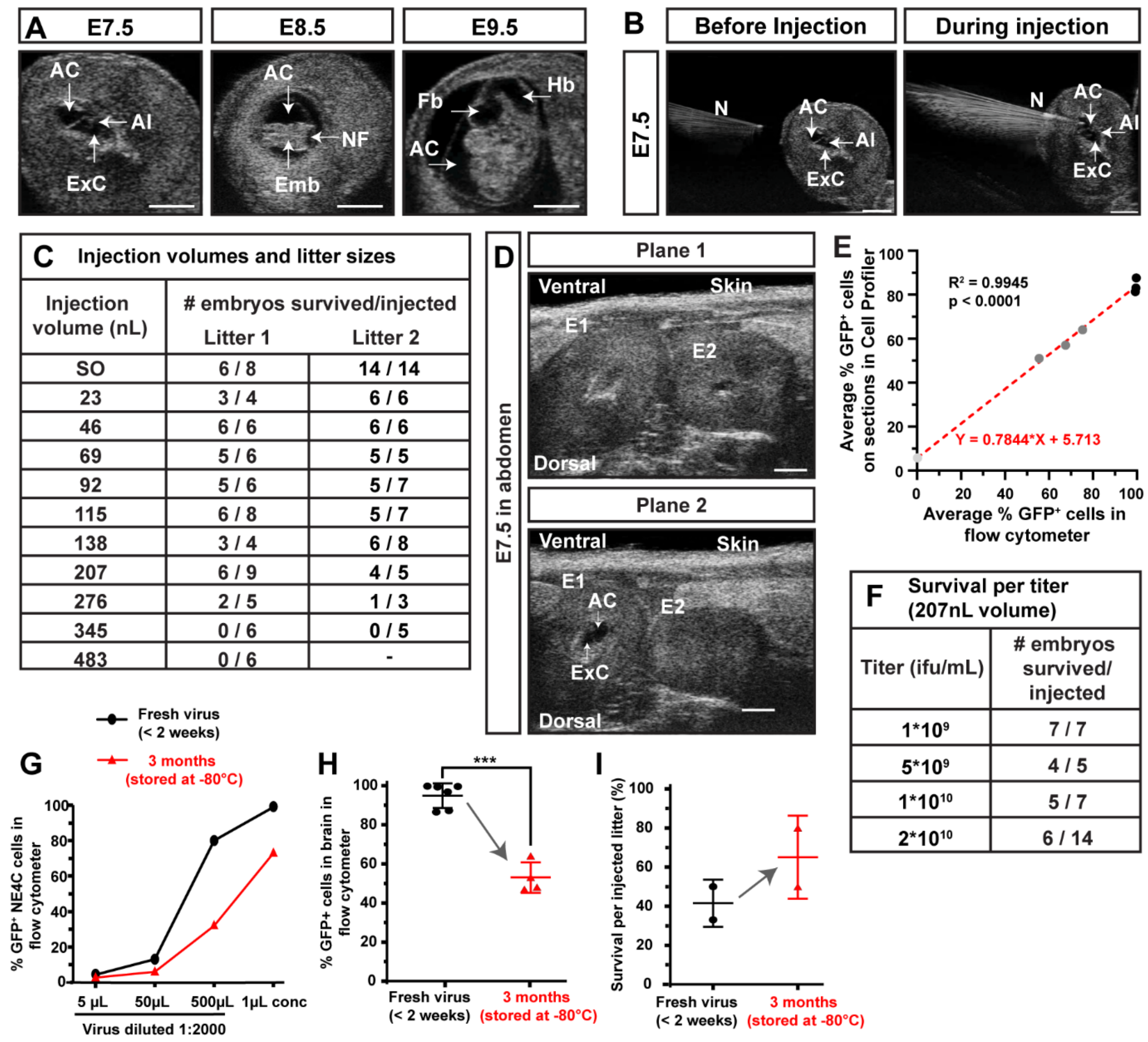


**C Injection volumes and litter sizes**

| Injection volume (nL) | # embryos survived/injected | |
|---|---|---|
| | Litter 1 | Litter 2 |
| SO | 6 / 8 | 14 / 14 |
| 23 | 3 / 4 | 6 / 6 |
| 46 | 6 / 6 | 6 / 6 |
| 69 | 5 / 6 | 5 / 5 |
| 92 | 5 / 6 | 5 / 7 |
| 115 | 6 / 8 | 5 / 7 |
| 138 | 3 / 4 | 6 / 8 |
| 207 | 6 / 9 | 4 / 5 |
| 276 | 2 / 5 | 1 / 3 |
| 345 | 0 / 6 | 0 / 5 |
| 483 | 0 / 6 | - |

**F Survival per titer (207nL volume)**

| Titer (ifu/mL) | # embryos survived/ injected |
|---|---|
| $1*10^9$ | 7 / 7 |
| $5*10^9$ | 4 / 5 |
| $1*10^{10}$ | 5 / 7 |
| $2*10^{10}$ | 6 / 14 |

**Fig. S1. Optimization parameters for NEPTUNE, Related to Figure 1.** (A) Neural development between E7.5-E9.5 is easily discerned by ultrasound, allowing identification of the amniotic cavity (AC), allantois (Al), and exocoelomic cavity (ExC) at E7.5; of the embryo (Emb), or neural folds (NF) at E8.5, and of the brain at E9.5 with an obvious forebrain (Fb) and hindbrain (Hb). (B) A sharp needle (N) well-aligned with the amniotic cavity ensures injection without puncturing the embryo or allantois at E7.5 Note that images from (A) and (B) show the same embryo at E7.5. (C) Summary table for data in Fig 1B, of volume optimization with each injected volume and the number of embryos that survived over total number injected in each litter. (D) Staging can be assessed prior to surgery, here ultrasound through the abdominal wall of the pregnant female at E7.5 to assess suitability of proceeding with the experiment. E1 = Embryo 1; E2 = Embryo 2 (E) Correlation of GFP+ cells detected either via Cell Profiler or flow cytometry, with the latter being slightly more sensitive. (F) Impact of viral titer on embryo survival: number of embryos that survived over total number injected for each titer. (G-I) Impact of long-term storage on viral titer *in vitro* in NE4C cells (G) and transduction efficacy *in vivo* (H). Virus was titrated directly after concentration and again after 3 months stored at -80°C (G). Transduction efficacy drops circa 50% (H) while survival per injected litter is slightly increased (I). Scale bars in all ultrasound images are 1 mm. Statistical analysis in (H): Two-tailed t-test, $p < 0.001$. Correlation analysis in E is two-sided Pearson correlation coefficient. $R^2$ and p-value are presented in the graph.

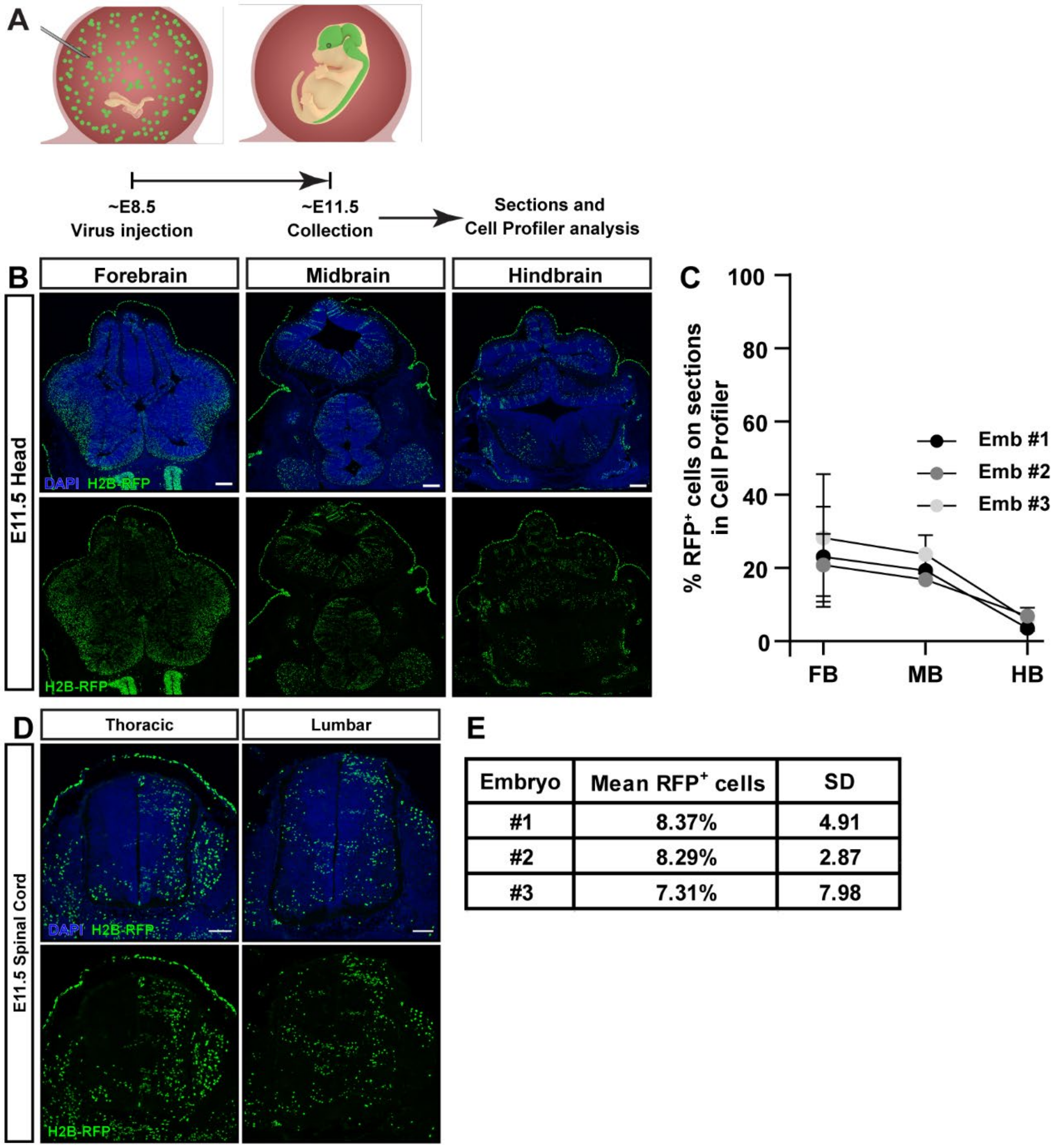


| Embryo | Mean RFP+ cells | SD |
|---|---|---|
| #1 | 8.37% | 4.91 |
| #2 | 8.29% | 2.87 |
| #3 | 7.31% | 7.98 |

**Fig. S2. *In utero* nano injection of virus at E8.5 results in low transduction efficacy and high variability across different brain regions, Related to Figure 1.** (A) Lentivirus was injected into the amniotic cavity around E8.5. Embryos were collected at E11.5, sectioned and number of transduced cells quantified via Cell Profiler. (B) Forebrain, Midbrain and Hindbrain are targeted with injections at E8.5. However, efficacy was much lower and was uneven across different brain regions, note for example columns of positive cells radiating from ventricular to marginal zone in Midbrain. (C) Cell Profiler quantification of GFP+ cells in three embryos in sections from forebrain (FB), midbrain (MB) and hindbrain (HB). Each dot represents the average +/- standard deviation of multiple sections per embryo per brain region (D,E) H2B-GFP+ cells in thoracic and lumbar spinal cord. Sections shown in D, and Table with quantification from three embryos shown in E. Average of all regions is presented for each embryo. Fewer than 10% of cells in spinal cord were targeted (E). Scale bars in B = 200µm and in D = 100µm.

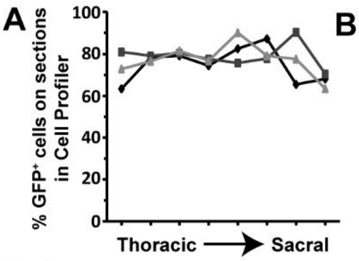


B

| | GFP⁺ cells in brain | GFP⁺ cells in spinal cord |
|---|---|---|
| Embryo # 1 | 87.7% | 74.7% |
| Embryo # 2 | 81.3% | 79.0% |
| Embryo # 3 | 83.0% | 77.3% |

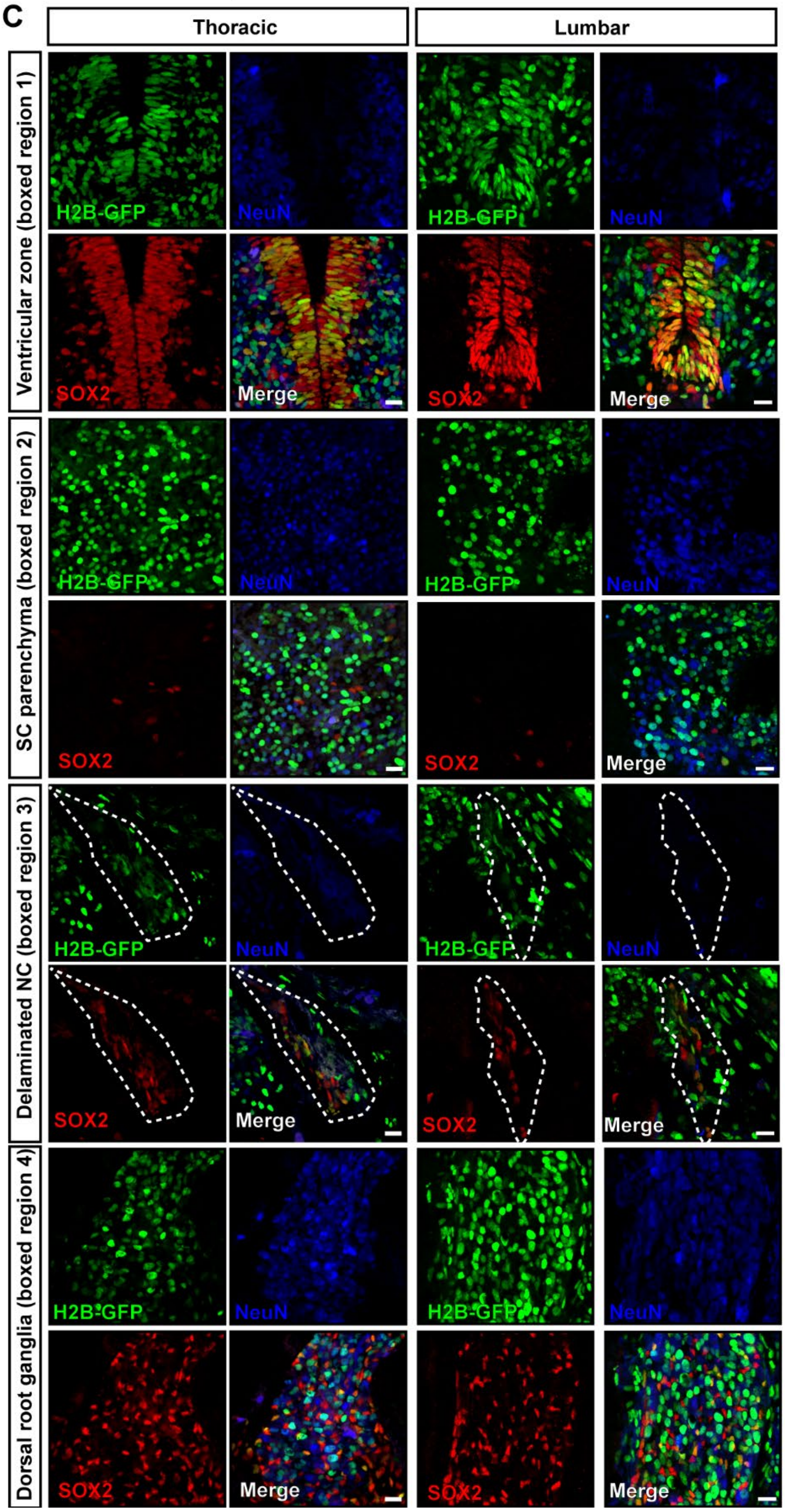


**Fig S3. *In utero* nano injection of virus at E7.5 targets the spinal cord evenly, Related to Figure 3.** (A) Cell Profiler results of GFP$^+$ cells detected on Spinal Cord sections from thoracic to sacral levels. Each dot represents quantification from one section of spinal cord. (B) Quantification of GFP+ cells using CellProfiler in brain and spinal cord from three embryos, injected at E7.5 and collected at E13.5, shows that spinal cord is consistently slightly less positive than brain. Each row represents quantification from brain and spinal cord from one embryo. Brain data is from Cell Profiler quantifications from brain halves also depicted in S1E as black dots. (C) Split channels of immunostainings presented in Fig 3B-E and G-J, for neural progenitors (SOX2+) and neurons (NeuN+) in spinal cord sections at the ventricular zone, in the parenchyma, delaminating neural crest and dorsal root ganglia in thoracic and lumbar section. Scale bars are 10µm.

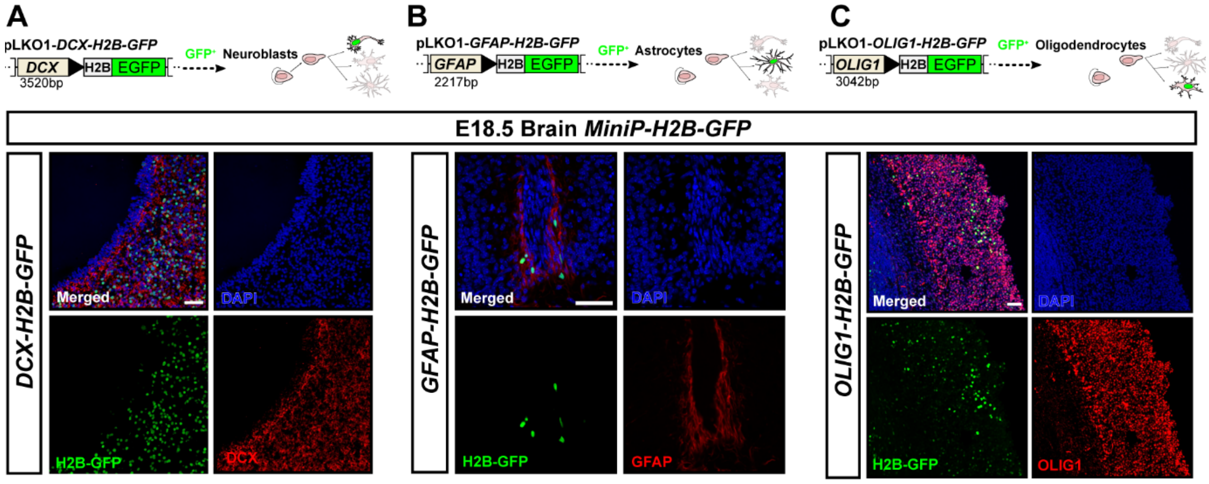


**Fig S4. Expression of MiniPromoter constructs in corresponding cell types at E18.5 in brain, Related to Figure 4.** (A) GFP in a *DCX-H2B-GFP* embryo overlaps with DCX in ventrolateral midbrain. (B) GFP in a *GFAP-H2B-GFP* embryo is exclusively expressed in GFAP+ astrocytes, shown here in indusium griseum. (C) GFP in an *OLIG1-H2B-GFP* embryo is present overlapping with OLIG1+ cells, but also overlaps with OLIG1- cells, shown here in dorsolateral cortex. Scalebars in all panels = 50 µm.

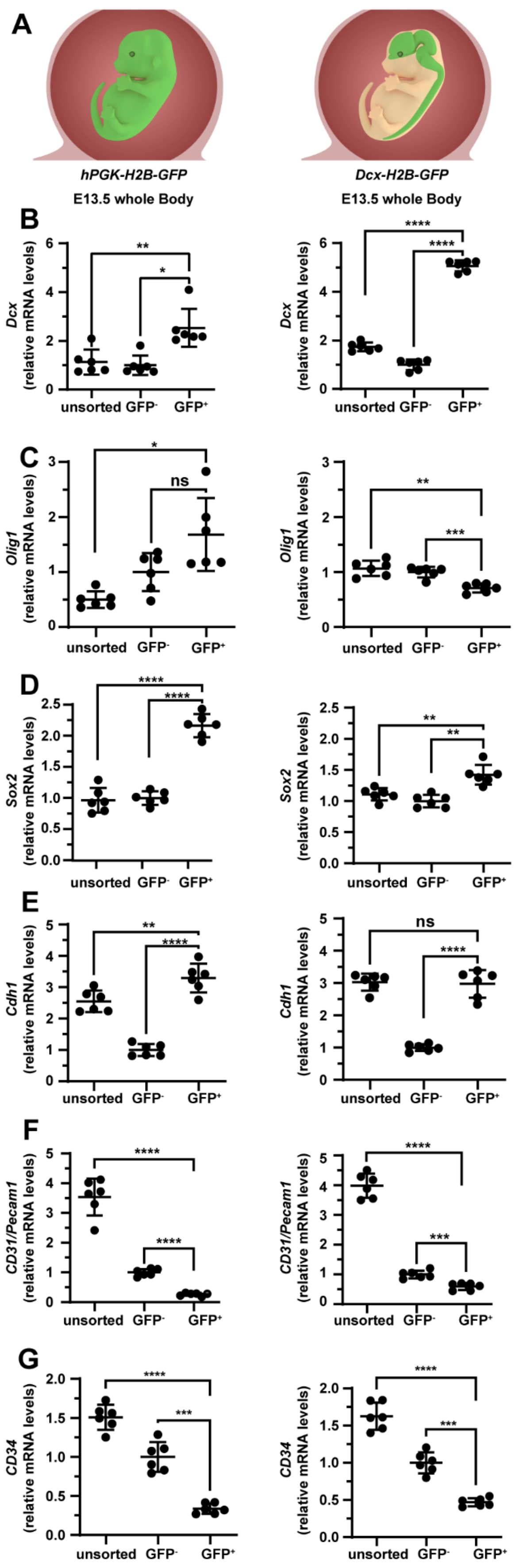


**Fig S5. Targeting of tissues and cells with *in utero* nano-injection at E7.5 with *hPGK-H2B-GFP* or *DCX-H2B-GFP,* Related to Figure 5.** (A) Schematic representation of embryos at E13.5, injected with either *hPGK-H2B-GFP* or *DCX-H2B-GFP* at E7.5. Whole bodies were collected and dissociated. GFP positive and negative cells were sorted for qPCR analysis, and pre-sorting mix of dissociated cells was included as a control. (B-G) qPCR for *Dcx* (B), *Olig1* (C), *Sox2* (D), *Cdh1* (E), *CD31* (F) and *CD34* (G) in *hPGK-H2B-GFP* embryos (at left) and *DCX-H2B-GFP* embryos (at right). mRNA expression levels are normalized to *Actb*. Differences in expression levels were analyzed with one-way ANOVA and Dunnett's multiple comparisons test. ns = not significant, * $p < 0.05$, ** $p < 0.01$, *** $p < 0.001$, **** $p < 0.0001$.

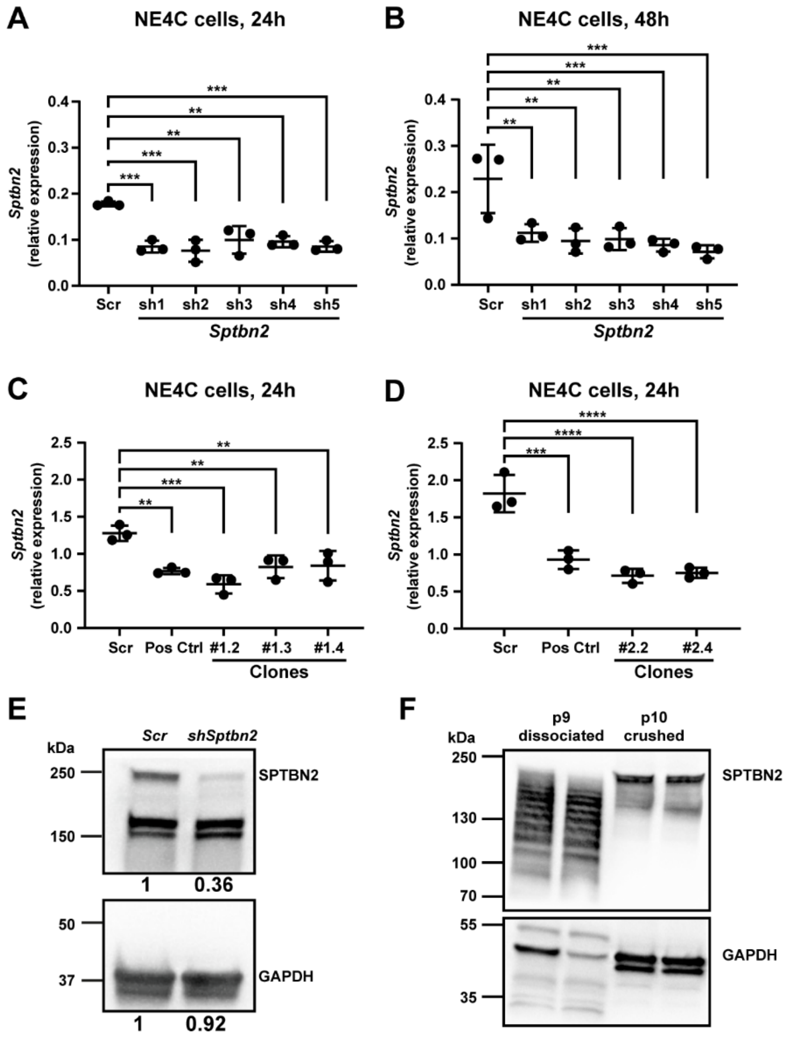


**Fig S6. Validation of *Sptbn2* shRNA in vitro, and western blot conditions for SPTBN2, Related to Figure 7.** NE4C cells were transfected with five different shRNAs, all targeting *Sptbn2* mRNA. (A) qPCR after 24h showed 50% knock down efficacy for all five constructs, which could still be observed at 48h post transfection (B). Each dot represents a biological replicate. Differences in expression levels were analyzed with one-way ANOVA and Dunnett's multiple comparisons test. ns = not significant, * $p < 0.05$, **$p < 0.01$, *** $p < 0.001$, **** $p < 0.0001$. (C and D) qPCR for *Sptbn2* mRNA in NE4C cells after transfection with different clones of *U6-shRNA-hPGK-H2B-GFP*. Knock down efficacy also at 50% reduction of mRNA levels compared to scrambled control. Each dot represents a technical replicate in one representative experiment. Differences in expression levels were analyzed with one-way ANOVA and Dunnett's multiple comparisons test. ns = not significant, * $p < 0.05$, **$p < 0.01$, *** $p < 0.001$, **** $p < 0.0001$. (E) Western Blot for SPTBN2 protein in NE4C cells, 1 week post-lentiviral transduction with either shRNA or scrambled control virus. Numbers below blots denote quantification of bands (top band for SPTBN2 corresponding to full-length SPTBN2). (F) Tissue dissociation disrupts SPTBN2 protein. Cerebellum from wild type P9 or P10 cerebellum were either dissociated for analysis or immediately frozen and crushed. Both SPTBN2 and GAPDH display multiple bands in dissociated cells, but a single band for SPTBN2 and the expected double band for GAPDH in frozen and crushed cerebellum.

Methods S1: Embryo partitioning for sectioning, related to STAR Methods Mangold et al

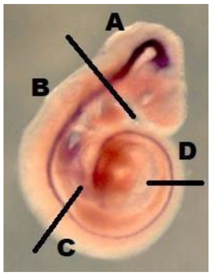


Fig Methods S1 – Partitioning of embryo for sectioning.
